# Applying the new multi-objective algorithms for the operation of a multi-reservoir system in hydropower plants

**DOI:** 10.1038/s41598-024-54326-z

**Published:** 2024-02-13

**Authors:** Syed Mohsen Samare Hashemi, Amir Robati, Mohammad Ali Kazerooni

**Affiliations:** Department of Civil Engineering, Islamic Azad University-Kerman Branch, Kerman, Iran

**Keywords:** Multi-objective evolutionary algorithm, Multi-reservoir systems, Karun basin, Hydrology, Engineering, Mathematics and computing

## Abstract

The optimal operation of the multi-purpose reservoir system is a difficult, and, sometimes, non-linear problem in multi-objective optimization. By simulating biological behavior, meta-heuristic algorithms scan the decision space and can offer a set of points as a group of solutions to a problem. Because it is essential to simultaneously optimize several competing objectives and consider relevant constraints as the main problem in many optimization problems, researchers have improved their ability to solve multi-objective problems by developing complementary multi-objective algorithms. Because the AHA algorithm is new, its multi-objective version, MOAHA (multi-objective artificial hummingbird algorithm), was used in this study and compared with two novel multi-objective algorithms, MOMSA and MOMGA. Schaffer and MMF1 were used as two standard multi-objective benchmark functions to gauge the effectiveness of the proposed method. Then, for 180 months, the best way to operate the reservoir system of the Karun River basin, which includes Karun 4, Karun 3, Karun 1, Masjed-e-Soleyman, and Gotvand Olia dams to generate hydropower energy, supply downstream demands (drinking, agriculture, industry, environmental), and control flooding was examined from September 2000 to August 2015. Four performance appraisal criteria (GD, S, Δ, and MS) and four evaluation indices (reliability, resiliency, vulnerability, and sustainability) were used in Karun's multi-objective multi-reservoir problem to evaluate the performance of the multi-objective algorithm. All three algorithms demonstrated strong capability in criterion problems by using multi-objective algorithms’ criteria and performance indicators. The large-scale (1800 dimensions) of the multi-objective operation of the Karun Basin reservoir system was another problem. With a minimum of 1441.71 objectives and an average annual hydropower energy manufacturing of 17,166.47 GW, the MOAHA algorithm demonstrated considerable ability compared to the other two. The final results demonstrated the MOAHA algorithm’s excellent performance, particularly in difficult and significant problems such as multi-reservoir systems' optimal operation under various objectives.

## Introduction

Due to numerous decision variables caused by the multiplicity of factors and depending on the time step required in exploitation (such as monthly, weekly, or daily), the optimum exploitation of water systems as an undeniable necessity is usually a large-dimension problem in complex optimization problems. The multiplicity of reservoirs with different purposes, including supply of downstream needs, electric energy production, and flood control, adds to the complexity of optimization operation problems.

Optimal exploitation requires formulating an objective function and then maximizing or minimizing it. A literature review shows that desired functions are usually diverse, complex, and have many extrema. As such, maximizing or minimizing them through linear and definite methods is impossible. Besides, objective functions have several variations^1,2^. The existence of local and multiple extrema in problems such as the exploitation of water systems has led to the development of meta-heuristic methods to optimize objective functions. The genetic algorithm (GA) was introduced in 1975 by Holland^3^ for an optimization process. In this algorithm’s first water resource applications, Esat^4^ used GA to examine four reservoir problems. Then, Oliveria and Loucks^5^ and Wardlaw and Sharif^6^ utilized this algorithm in water resources problems.

Since it is essential to simultaneously optimize several competing objectives and consider relevant constraints as the main problem in many optimization problems, researchers have increased optimization algorithms’ capability in solving multi-objective problems by developing complementary multi-objective algorithms. Examples include the strength Pareto evolutionary algorithm (SPEA)^7^, Pareto envelope-based selection algorithm (PESA)^8^, non-dominated sorting genetic algorithm-II (NSGA-II)^9^, multi-objective particle swarm optimization (MOPSO)^10^, multi-objective water cycle algorithm (MOWCA)^11^, and multi-objective grey wolf optimizer (MOGWO)^12^.

The artificial hummingbird algorithm (AHA), developed by Zhao et al.^13^, is a novel algorithm designed to enhance exploration and exploitation capabilities. The multi-objective version of the AHA was used and adapted to optimal exploitation problems for water systems in the multi-reservoir system of the Karun River due to AHA’s greater ability to explore and extract than standard algorithms; besides, it was necessary to obtain optimization algorithms that suited the complexity of multi-reservoir and multi-objective water systems (Karun 1, Karun 3, Karun 4, Gotvand Olia, and Masjed-e-Soleyman dams). Additionally, the MOAHA multi-objective algorithm was examined in Karun's multi-objective-multi reservoir problems in accordance with different objectives, including supply and demand, electric energy production, and flood management.

The development of algorithms with this level of strength was prompted by the need to solve problems that consider multiple objectives concurrently. The multi-objective water cycle algorithm (MOWCA), multi-objective gray wolf algorithm (MOGWO), multi-objective moth swarm algorithm (MOMSA), and other algorithms have all been developed for this purpose. A novel multi-objective artificial hummingbird algorithm (MOAHA) was developed in the current research to address multi-objective problems. The extraordinary performance of the developed algorithm in solving complicated and large-scale problems, such as problems of exploitation of multi-objective reservoir systems, is one of its key advantages over other multi-objective algorithms currently in use. Furthermore, its performance was compared with two new multi-objective algorithms, the multi-objective moth swarm algorithm (MOMSA) and the multi-objective material generation algorithm (MOMGA).

### A review of the literature on reservoir operation

Chen^14^ used a genetic method for reservoir multi-objective optimization. This optimization was done for the long-term operation of the reservoir. The results showed that the genetic algorithm was useful, especially in non-linear systems. Chen et al.^2^ used another type of genetic algorithm to optimize reservoir consumption based on the objective functions of maximum power plant production and water storage. The feature of the latter algorithm was preventing premature convergence during the normal genetic algorithm process. Schardong et al.^15^ used a multi-objective evolutionary approach for suitable reservoir exploitation. Using the multi-objective algorithm of differential evolution (MODE), they made optimal use of part of a complex reservoir system that supplied water for about 20 million people in urban areas. The studied objectives included minimizing shortages (the difference between demand and allocated water), maximizing water quality, and minimizing the pumping cost. The developed algorithm (MODE) was also compared with the multi-objective genetic algorithm and non-dominant sorting (NSGA-II).

The proposed algorithm (MODE) provides a closer convergence to the actual Pareto front than the NSGA-II algorithm. Qaderi et al.^16^ exploited the reservoir system using a water cycle algorithm (WCA). First, they used the water cycle algorithm to solve the problems of 4 and 10 benchmark reservoirs and then used this algorithm to solve the problem of exploiting the reservoir system of the Gorgan River basin. Their results showed that water cycle algorithm provided a suitable estimate with 0.5 and 1% difference compared to the global optimum, respectively, in problems of 4 and 10 reservoirs. They also reported the superiority of the water cycle algorithm compared to GA and PSO. Liu et al.^17^ used flood control and electric energy production functions to exploit the storage dam of three valleys in China optimally. For this purpose, they utilized the progressive optimality algorithm (POA) to determine the optimal utilization of overflows and smooth support vector machine model (SSVM) to exploit real-time reservoirs. The studied methods for the short-term operation of the reservoir reduced the risk of flooding and increased hydropower production during the flood season.

Afshar and Hajiabadi^18^ suggested a novel technique known as parallel cellular automata (PCA) to optimize the performance of a multi-objective reservoir. They used the two opposing objectives of producing energy and providing water as their primary function. Different time scales of 60, 120, 240, and 480 months were considered to test the operation of the proposed algorithm, and the results were compared with those of the NSGA-II algorithm. The proposed method was superior to the NSGA-II algorithm by increasing the problem scale and requiring less computing time. Takada et al.^19^ developed a method to create the best operational command curves for the Dao Teing Reservoir, one of the largest multi-purpose reservoirs in Vietnam. They determined command curves via a modified complex evolutionary method. Experimental findings demonstrated that the suggested optimization strategy efficiently searched for the best command curves.

Shen et al.^20^ proposed a novel large-scale energy transmission optimization model in 2020 to support China's interprovincial hydropower system (IHS) operation. This multi-objective optimization model was developed for the monthly use of IHS, with peak-shaving daily requirements in mind. Peak-shaving significantly increased with a relatively small decrease in energy production according to the obtained Pareto solutions that examined the interdependence between peak-shaving and energy production objectives. To identify compatible command curves, Thongwan et al.^21^ optimized a simulation model of Thailand's Ubolrat reservoir using conditional tabu search algorithms (CTSA) and conditional genetic algorithms (CGA). Based on the findings, the multi-objective command curves created by CGA were more successful than CTSA in reducing the frequency of floods and drought conditions in the future. Zhang et al.^22^ improved the multi-objective flame-butterfly optimization algorithm based on R-dominance to solve the multi-objective optimal exploitation model of reservoirs with power production, environmental protection, and navigation objectives (R-IMOMFO). Based on its comparisons with five other algorithms, the R-IMOMFO algorithm yielded a group of solutions with a strong distribution and excellent convergence to the problem of the optimal exploitation of reservoirs. The findings of the exploitation revealed an apparent conflict between the demand for navigation and the environment. Liu et al.^23^ used the NSGA-II algorithm and the lion pride algorithm (LPA) to maximize the use of bi-objective reservoirs. The LPA algorithm ran for bi-objective optimization problems about two to four times faster than NSGA-II because it performed better in convergence and divergence than NSGA-II.

Ahmadianfar et al.^24^ introduced an enhanced differential evolution (EDE) algorithm to extract the optimal policies of hydropower multi-reservoir systems. The EDE had powerful global ability and faster convergence than the original DE. Kumar and Yadav^25^ proposed the self-adaptive multi-population multi-objective Jaya algorithm (SAMP-MOJA) to extract multi-purpose reservoir operation policies. The results were compared with those of the MOJA and the MOPSO algorithms. SAMP-JA outperformed JA, the PSO, and IWO regarding maximum hydropower generation and the algorithm convergence rate. Mansouri et al.^26^ employed an improved version of the fuzzy multi-objective PSO algorithm (f-MOPSO) to optimize the reservoir operation under climate change. The results indicated the superior performance of f-MOPSO to the NSGA-II in meeting the water demands and holding the reservoir storage sustainable. Nguyen^27^ used the optimization approach to determine the optimal water discharge scenario in the operating multi-purpose reservoirs in the Red River basin. There was increasing economic benefit from saved water and hydropower generation during peak hours.

Furthermore, the economic value added by the power generation of three reservoirs was about 401.7 billion VND. Fang et al.^28^ developed an accelerated version of gradient-based optimization (AGBO) to solve a complex multi-reservoir hydropower system. The optimal results showed that the method was superior to the other advanced optimization algorithms for maximizing the load demands in the hydropower system.

### A review of the literature on the Karun basin

Sharifi et al.^29^ developed a new fitness-distance-balance selection method in the moth swarm algorithm (FDB-MSA) to optimize the hydropower generation of a real five-reservoir system along Karun River. The results revealed the superiority of FDB-MSA to GA and PSO; the smallest difference (3.41%) was observed between nominal and optimal power generation compared to the PSO (6.58%) and GA (33.89%). Also, Sharifi et al.^30^ investigated the capability of 14 new robust evolutionary algorithms (EAs) in optimizing energy generation from the Karun-4 hydropower reservoir in the Karun basin. The overall results indicated the promising capability of some EAs for optimal operation of hydropower reservoirs. Ahmadianfar et al.^31^ aimed to extract nonlinear operating rules of the great Karun basin multi-reservoir systems; for this purpose, they developed a self-adaptive teaching learning-based algorithm with differential evolution (SATLDE). The SATLDE achieved superior precision and reliability than other methods. The results also revealed that SATLDE increased the total power generation by up to 23.70% compared to other advanced optimization methods. Vahabzadeh et al.^32^ developed a comprehensive energy simulation model (CESNeX) for energy-water-food nexus system analysis for the great Karun basin water resources system. The results indicated the desirable performance of the proposed simulator model in the Karun basin. Mostaghimzadeh et al.^33^ proposed a long lead time forecast model applying an ensemble approach to manage the great Karun multi-reservoir system. The proposed model significantly improved the final results. Ahmadianfar et al.^34^ developed the influential flower pollination algorithm (IFPA) for the real-world five-reservoir hydropower system in the Karun basin. The IFPA achieved better total power production compared to other algorithms, including adaptive guided differential evolution (AGDE), composite DE (CODE), hybridizing sum-local search optimizer (HSLSO), improved teaching learning-based optimization (ITLBO), self-adapting control parameters in DE (jDE), self-adaptive TLBO (SATLBO), and a multi-strategy hybrid of DE and particle swarm optimization (MS-DEPSO).

The Karun multi-purpose multi-reservoir system is the largest system of cascade reservoirs in Iran. It is one of Iran's most important and strategic reservoir systems and provides more than 90% of the country's renewable hydropower energy. The main advantage of the Karun system is that it is multi-purpose (water supply, energy generation, and flood control) and has five cascade reservoirs (Karun-4, Karun-3, Karun-1, Masjed-e-Soleyman, and Gotvand Olia dams), which make it a complex, large-scale optimization problem.

Given the above, the operation of large-scale multi-reservoir multi-objective systems is complicated, and we need robust algorithms to obtain the best operation policies for such problems. The best operational policy yields the minimum total deficit during the operational period while considering power generation and flood control objectives simultaneously. The powerful multi-objective algorithms should solve this problem as an integrated model.

To the best of the authors' knowledge, limited studies have applied the MOAHA algorithm for water resources planning and management in limited studies, particularly for the optimal operation of multi-reservoir system. Therefore, the MOAHA multi-objective algorithm was used for optimal multi-objective exploitation of the multi-reservoir system of Karun, one of the most important water resources systems in Iran. Besides, its performance was compared with two new multi-objective algorithms, MOMSA and MOMGA.

## Materials and methods

**Figure 1:**
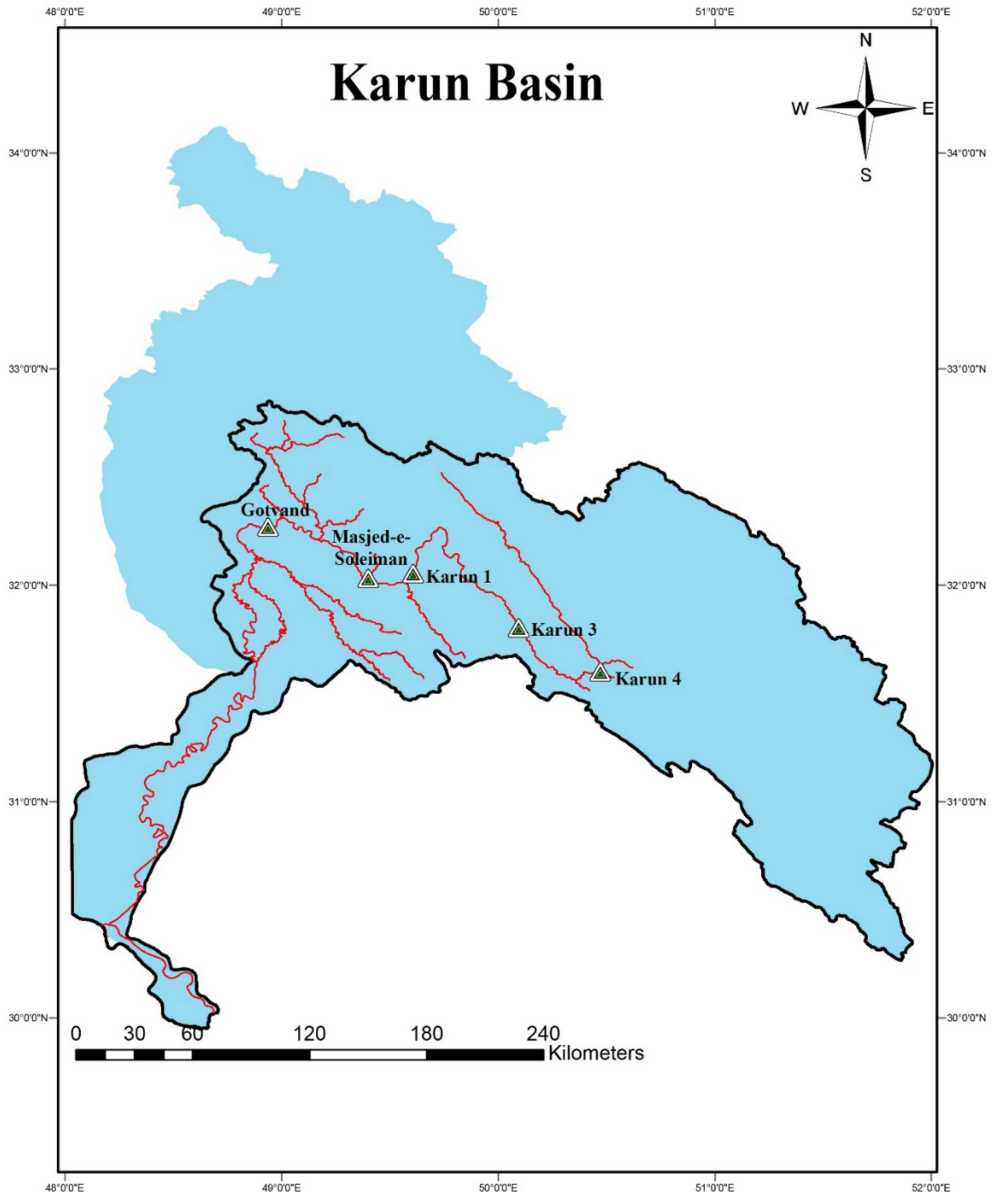
The location of each dam in the Karun basin.

There are four significant storage dams (Gotvand Olia, Karun 1, Karun 3, and Karun 4) and a hydropower dam (Masjed-e-Soleyman) on the Karun River in the Karun basin, one of the richest basins in Iran: Due to 32 power plant units with a combined capacity of over 8500 MW (Table 1) for each of the dams, the system has a significant impact on supplying hydroelectric energy. With a nominal annual electricity output of 18,248.7 GW, it provides more than 90% of the nation's total hydroelectric energy (Dezab Consulting Engineering Co., 2019). The 67,257 km^2^ Karun watershed is located between 48° 10′ and 52° 30′ east longitude and 30° 20′ and 34° 05′ north latitude. Figure 1 depicts the Gotvand Olia, Masjed-e-Soleyman, Karun 1, Karun 3, and Karun 4 dams, which are all situated on the main branch of the Karun River in succession, along with the location of the Karun basin.

**Table 1:** Details of the hydroelectric infrastructure at Gotvand Olia, Masjed-e-Soleyman, Karun 4, Karun 3, and Karun 1^35^.

Parameter	Unit	Karun 4	Karun 3	Karun 1	Majed-e-Soleiman	Gotvand
Number of power plant units	Number	4	8	8	8	4
Power of each unit	Megawatt (MW)	250	250	250	250	375
The total capacity of power plant units	Megawatt (MW)	1000	2000	2000	2000	1500
Average annual energy production	Gigawatt hours (GWH)	2190.6	3890.8	3951.9	3959.8	4255.6
Efficiency of hydropower generation	%	92	92.4	90	92	93
Perfomance coefficient	%	25	22	23	23	32
Power plant tail-water level	Meters above mean sea level (masl)	840	665	365	240	75

Each hydropower plant comprises several independent power production units with a specific production capability (PPC). Each hydroelectric power facility also has a specific production efficiency to indicate its effectiveness. The quantity of electricity generated by each power plant is based on the efficiency coefficient over a particular time frame. The percentage of times a power plant runs at maximum efficiency throughout an operational period is denoted by this coefficient. The Karun basin’s electric reservoir system has proven successful over several months of operation, and each power plant can produce energy for at least 5–8 h every day for at least 1 month. Table 1 lists information about the electric power plants in the Karun River basin investigated for this study^35^.

The specified system’s quantity and quality of exploitation are subject to stochastic influences, including the input flow to each reservoirs and multiple uses in the main electricity sector production of Iran, all of which multiply the importance of planning and justify any effort to optimize the objectives of the mentioned system, including hydropower generation.

### Utilized data

In this study, 180-month time-series data, from 2000 to 2015, were used for the optimal operation of water resources in the Karun basin. The utilized data include net evaporation, river inflow, and total demands in the Gotvand Olia, Masjed-e-Soleyman, Karun 1, Karun 3, and Karun 4 dams (Table 2).

**Table 2:** Utilized data range in the study area.

Dams	Data range	Net evaporation (mm)	Average river inflow (MCM)	Total water demands (MCM)
Gotvand Olia	Minimum	56.4	1.8	525.7
	Mean	182.5	175.6	725.7
	Maximum	306.5	999.3	941.9
Masjed-e-Soleyman	Minimum	45.4	0.7	0.5
	Mean	167.4	166	0.6
	Maximum	302.5	918.8	0.7
Karun 1	Minimum	46.3	92.4	0.4
	Mean	158.9	306	0.6
	Maximum	292.9	2304.2	1.2
Karun 3	Minimum	43.5	17.1	0.2
	Mean	148.8	139.8	0.3
	Maximum	271.9	589.8	0.5
Karun 4	Minimum	42.4	84.7	0.1
	Mean	143.8	377.5	0.4
	Maximum	257.5	1503	1.2

### Problems with multi-objective optimization

While constrained by an MOP, multiple-objective functions must be minimized (or maximized) concurrently^36^. Therefore, MOP can be explained as follows:

1 $$Minimal\;function f\left( {\vec{x}} \right) = \left[ {f_{1} \left( {\vec{x}} \right), \ldots ,f_{i} \left( {\vec{x}} \right), \ldots , f_{n} \left( {\vec{x}} \right)} \right] \;\vec{x} \in R^{d}$$

2 $${\text{Limitations }}\quad \left\{ {\begin{array}{*{20}l} {g_{i} \left( {\vec{x}} \right) \le 0} \hfill & {i = 1, \ldots , P} \hfill \\ {h_{j} \left( {\vec{x}} \right) \le 0} \hfill & {i = 1, \ldots , q} \hfill \\ \end{array} } \right.$$

3 $${\text{Low}} \le \vec{x} \le up\quad {\text{Low}} \in R^{d} ,\quad {\text{up}} \in R^{d}$$

where $f_{i} \left( {\vec{x}} \right)$ is the i-th singular objective function, R^d^ is the d-dimensional solution space, g_i_ and h_i_ are the i-th equal and unequal constraints, respectively.

In single-objective optimization, it is simple to compare various solutions using interface functions, and algorithms can only present one solution as the overall best solution. However, the interface operators lose their effectiveness in multi-objective optimizations because it is challenging to evaluate solutions with many objectives as the objectives contend with one another. It is essential to locate another interface operator for comparison with all objectives. In this instance, Pareto's dominance of the topic resulted in sensible solutions that weigh several objectives. The key definitions provided by Pareto for MOP minimization are listed below.

#### Definition 1

(Pareto dominance^37^) vector $\vec{x}$ dominates vector $\vec{y}$ (denoted as $\vec{x} \le \vec{y}$) if:

4 $$\forall i \in \left\{ {1, \ldots ., m} \right\}, f_{i} \left( {\vec{x}} \right) \le f_{i} \left( {\vec{y}} \right)\, and\; \exists j \in \left\{ {1, \ldots . , m} \right\}, f_{i} \left( {\vec{x}} \right) \le f_{i} \left( {\vec{y}} \right).$$

#### Definition 2

(Pareto optimal) If and only if a strategy $\vec{x}$ is considered Pareto optimal:

5 $$\left\{ {\neg \exists \vec{y} \in R^{d} |\vec{y} < \vec{x}} \right\}.$$

#### Definition 3

(Pareto optimal set) The definition of the Pareto optimum set (PS) is as follows:

6 $$PS = \left\{ {\vec{x},\vec{y} \in R^{d} |\neg \exists \vec{y} < \vec{x}} \right\}.$$

#### Definition 4

(Pareto optimal front) This is how the Pareto optimum front (PF) is described:

7 $$PF = \left\{ {f\left( {\vec{x}} \right)|\vec{x} \in PS} \right\}.$$

**Figure 2:**
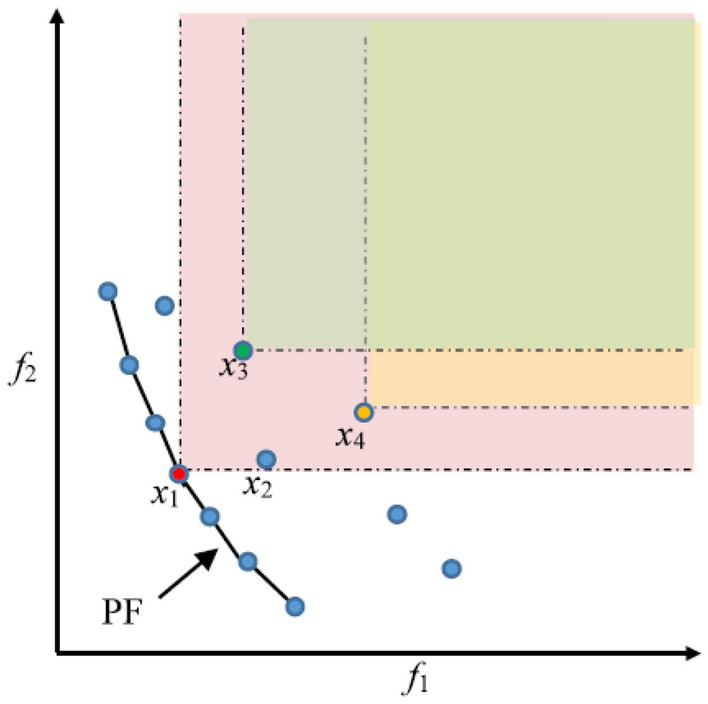
Schematic diagram of the relationship between dominant parameters in MOPs.

When dealing with MOPs, finding a PS rather than a singular optimal solution is an obvious requirement. A typical technique for storing and recovering PS in the optimizer process creates an external archive with a particular number. Figure 2 illustrates the relationship between the MOPs’ main ratios. In Fig. 2, the continuous line represents PF, and x_1_ is the PF's Pareto-optimal solution. x_1_ is better than x_2_, x_3_, and x_4_. Since x_3_ has a higher value for f_2_ and a smaller value for f_1_ than x_4_, x_3_ and x_4_ do not take precedence over each other.

### Standard deviation (distance from the mean)

**Figure 3:**
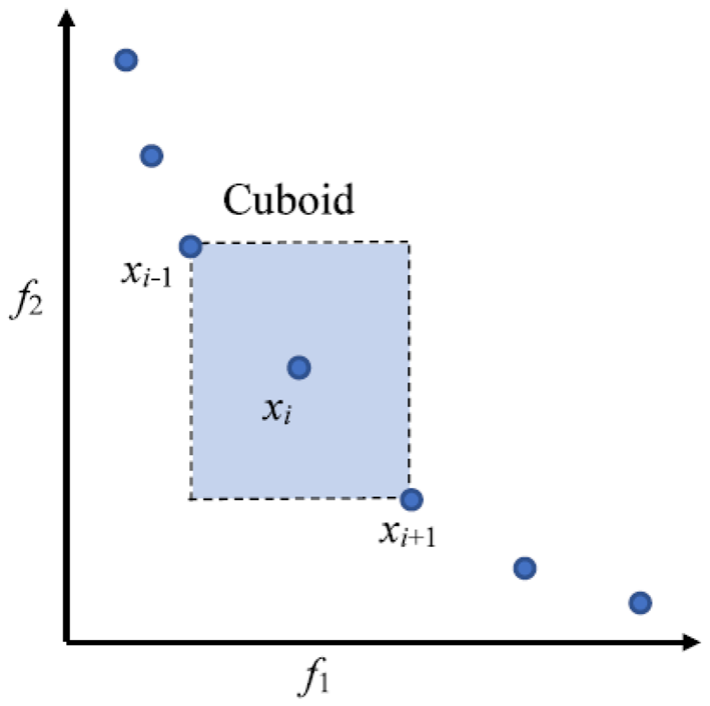
Crowding distance of two-objective solutions.

The standard deviation calculates the density of non-dominant solutions near the initial solution. MOPs are commonly used to maintain the distribution of solutions obtained in PF^9^. Based on the fitness values of each objective function, all of the solutions in the set of non-dominant solutions are arranged in ascending order. The average length of the rectangle sides for each solution, x_i_, is determined by the neighboring solutions x_i−1_ and x_i+1_. These numbers stand for the solution xi's mean standard deviation. It is typical to assume that the distance from the set of solutions with the minimum and maximum values of the objective function is infinite. A greater range of solutions is possible at a greater distance from the density of solutions. Figure 3 displays the separation from data congestion with two objectives.

Random archiving^38^, grid archiving^10^, and *ε*-Pareto archiver^39^ are some popular techniques for maintaining external archives. The random archiver randomly selects and removes the non-dominated solutions from the archive. The grid archiver partitions the target space into numerous hypercubes, and each hypercube saves the non-dominated solutions. Then, a preset number of the solutions are eliminated to distribute the remaining ones equally. A hypercube with two or more non-dominated solutions is archived using the *ε*-Pareto algorithm, but only the solution closest to the hypercube origin is kept. The value of *ε* determines how many hypercubes are broken up into the objective space.

### Artificial hummingbird algorithm (AHA)

Zhao et al.^13^ developed the bio-based algorithm known as AHA to handle single-objective optimization problems. It can be categorized as a swarm algorithm. This program was created based on how hummingbirds naturally forage. Three foraging behaviors—guided, territorial, and migratory—are modeled by AHA during the optimization process. Axial, diagonal, and omnidirectional are the three types of flight mimicked by foraging behaviors. A visit table also imitates how hummingbirds use their memories to select their preferred food sources. The key components in AHA are briefly described below^13^.

### Multi-objective artificial hummingbird algorithm (MOAHA)

MOAHA was first proposed by Zhao et al.^40^ for MOPs. Three major components are integrated into MOAHA as follows to perform multi-objective optimization effectively:

1. An external archive is introduced into MOAHA to save a fixed number of optimal non-dominated solutions during iterations.
2. A dynamic elimination-based crowding distance (DECD) method is employed to efficiently maintain the archive during the entire optimization process, aiming to improve the solution diversity significantly.
3. A solution update mechanism based on NDS is developed and used to refine non-dominated solutions in the solution update phase, remarkably contributing to solutions’ convergence.

### Solution update strategy based on NDS

In AHA, in each iteration, after performing guided foraging or territorial foraging, the current solution is replaced with the candidate solution if the fitness value of the candidate solution is better than that of the current solution, or the current solution will remain unchanged. However, in MOAHA, dominance relation with many objectives makes solution updating complicated. The selection of solution update strategy will directly affect convergence towards the true PF.

In MOAHA, a solution update strategy based on NDS is proposed. In this strategy, all solutions are sorted by the NDS approach; solutions dominated by fewer solutions have a higher dominance hierarchy, and all solutions are assigned different ranks according to their non-dominated levels.

**Figure 4:**
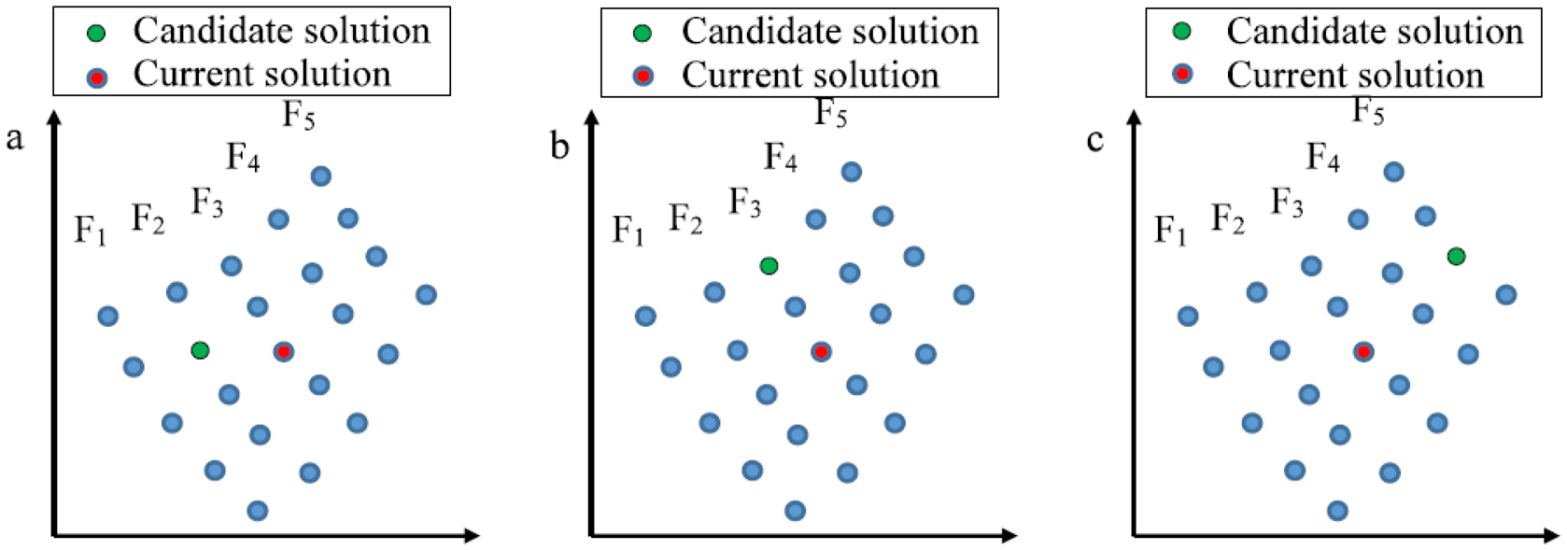
(**a**) The front on which the candidate solution lies is better than the front on which the current solution lies, (**b**) both the candidate solution and current solution belong to the same front, and (**c**) the front on which the candidate solution lies is worse than the front on which the current solution lies.

1. When the front on which the candidate solution of the ith individual lies is better than the front on which the current solution of the ith individual lies, the current solution of the ith individual is replaced by its candidate solution. In Fig. 4a, the candidate solution belongs to F_2_ front, the current solution belongs to F_3_ front, and the F_2_ front is better than the F_3_ front. In this case, the candidate solution will replace the current solution.
2. When both the candidate and current solutions of the *i*th individual belong to the same front, there is a probability of 50% to choose between the two to update the current solution of the *i*th individual with the candidate solution. Figure 4b depicts this case.
3. When the front on which the candidate solution of the *i*th individual lies is worse than the front on which the current solution of the *i*th individual lies, the current solution will be retained for the next iteration. This case is described in Fig. 4c. This solution update strategy is defined as follows:
  8 $$x_{i} \left( {t + 1} \right) = \left\{ {\begin{array}{*{20}l} {v_{i} \left( {t + 1} \right)} \hfill & {v_{i} \in F_{p} , x_{i} \in F_{q} \;and\;p < q} \hfill \\ {\left\{ {\begin{array}{*{20}l} {v_{i} \left( {t + 1} \right)} \hfill \\ {x_{i} \left( t \right)} \hfill \\ \end{array} } \right.} \hfill & {\begin{array}{*{20}l} {v_{i} \in F_{p} , x_{i} \in F_{q} ,\quad p = q\;and\;rand < 0.5} \hfill \\ {v_{i} \in F_{p} , x_{i} \in F_{q} ,\quad p = q\;and\;rand \ge 0.5} \hfill \\ \end{array} } \hfill \\ {x_{i} \left( t \right) } \hfill & {v_{i} \in F_{p} , x_{i} \in F_{q} ,\quad and\;p > q} \hfill \\ \end{array} } \right.$$

where F_p_ is the pth front in NDS.

### Dynamic elimination-based crowding distance (DECD)

**Figure 5:**
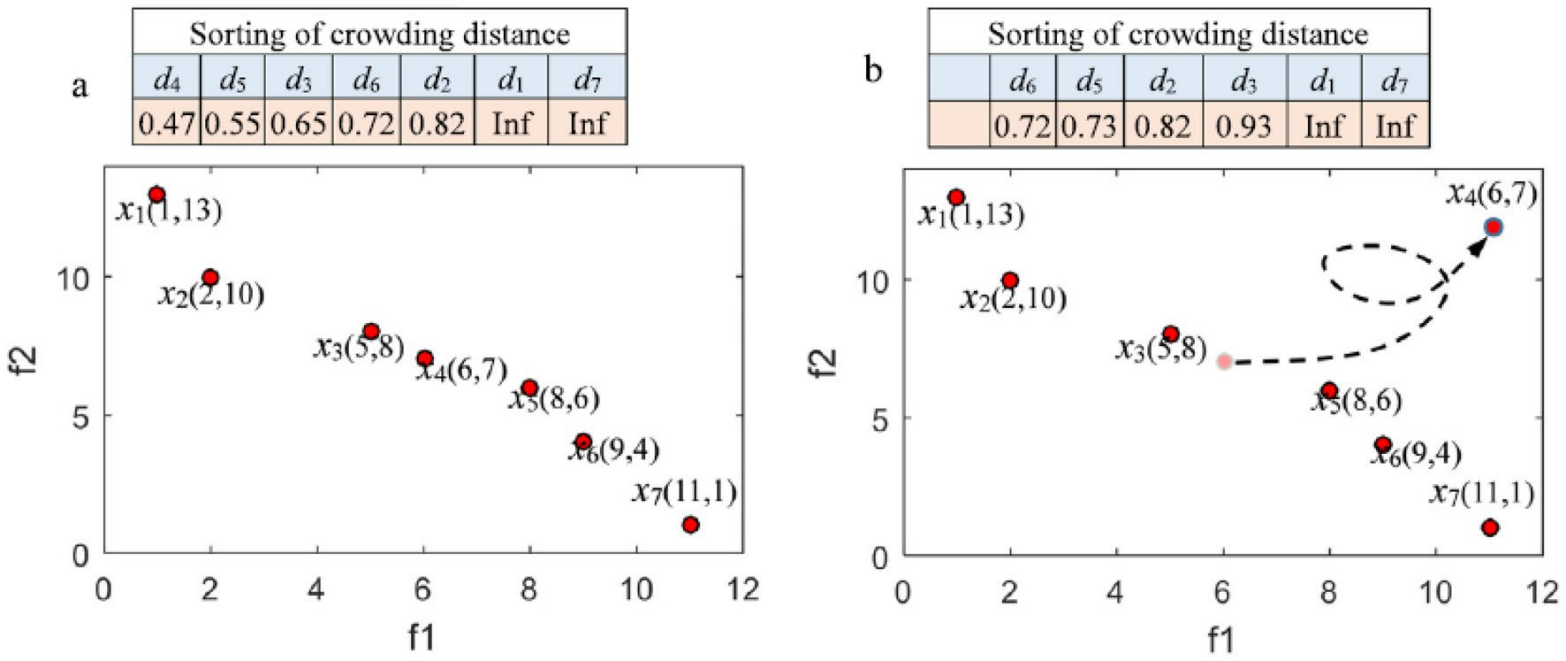
(**a**) Sorting of crowding distance before solution × 4 is removed, and (**b**) sorting of crowding distance after solution × 4 is removed.

The crowding distance is an effective parameter-free method to enhance the diversity of solutions^9^, so it is frequently employed in many MOAs to maintain a fixed-size external archive by removing excessive solutions with smaller crowding distance. However, whenever a solution is removed, the sorting of the crowding distance of the remaining solutions will be changed. Then, the next solution to be removed in terms of the initial crowding sorting may not be the one with current crowding sorting, which will reduce solution diversity in the external archive. Figure 5 displays the sorting change of the crowding distance before and after a solution is removed. Evidently, sorting the subsequent crowding distance is changed when the solution with the smallest crowding distance (*x*_4_) is removed. The next solution to be removed will be *x*_5_ according to the original sorting of the crowding distance in Fig. 5a.

Nevertheless, *x*_6_ should be removed according to the updated sorting of crowding distance in Fig. 5b. The archiving procedure based on crowding distance is ineffective in maintaining the sustainable diversity of PS. Thus, the external archive with DECD is proposed. When the solution with the current minimal crowding distance in PS is removed, only the crowding distances of the immediately adjacent solutions to remove the solution must be updated, and the crowding distances of the rest of the solutions do not change.

Before the *i*th solution with the current smallest crowding distance is removed, the crowding distances of the last and next solutions adjacent to the *i*th solution are given as follows, respectively:

9 $$\left\{ {\begin{array}{*{20}l} {d_{k} \left( {x_{i - 1} } \right) = \frac{{\left| {f_{k} \left( {x_{i} } \right) - f_{k} \left( {x_{i - 2} } \right)} \right|}}{{\max \left( {f_{k} } \right) - {\text{min}}\left( {f_{k} } \right)}}} \hfill \\ {D\left( {x_{i - 1} } \right) = \mathop \sum \limits_{k = 1}^{m} d_{k} \left( {x_{i - 1} } \right)} \hfill \\ \end{array} } \right.$$

10 $$\left\{ {\begin{array}{*{20}l} {d_{k} \left( {x_{i + 1} } \right) = \frac{{\left| {f_{k} \left( {x_{i} } \right) - f_{k} \left( {x_{i + 2} } \right)} \right|}}{{\max \left( {f_{k} } \right) - {\text{min}}\left( {f_{k} } \right)}}} \hfill \\ {D\left( {x_{i + 1} } \right) = \mathop \sum \limits_{k = 1}^{m} d_{k} \left( {x_{i + 1} } \right)} \hfill \\ \end{array} } \right..$$

**Figure 6:**
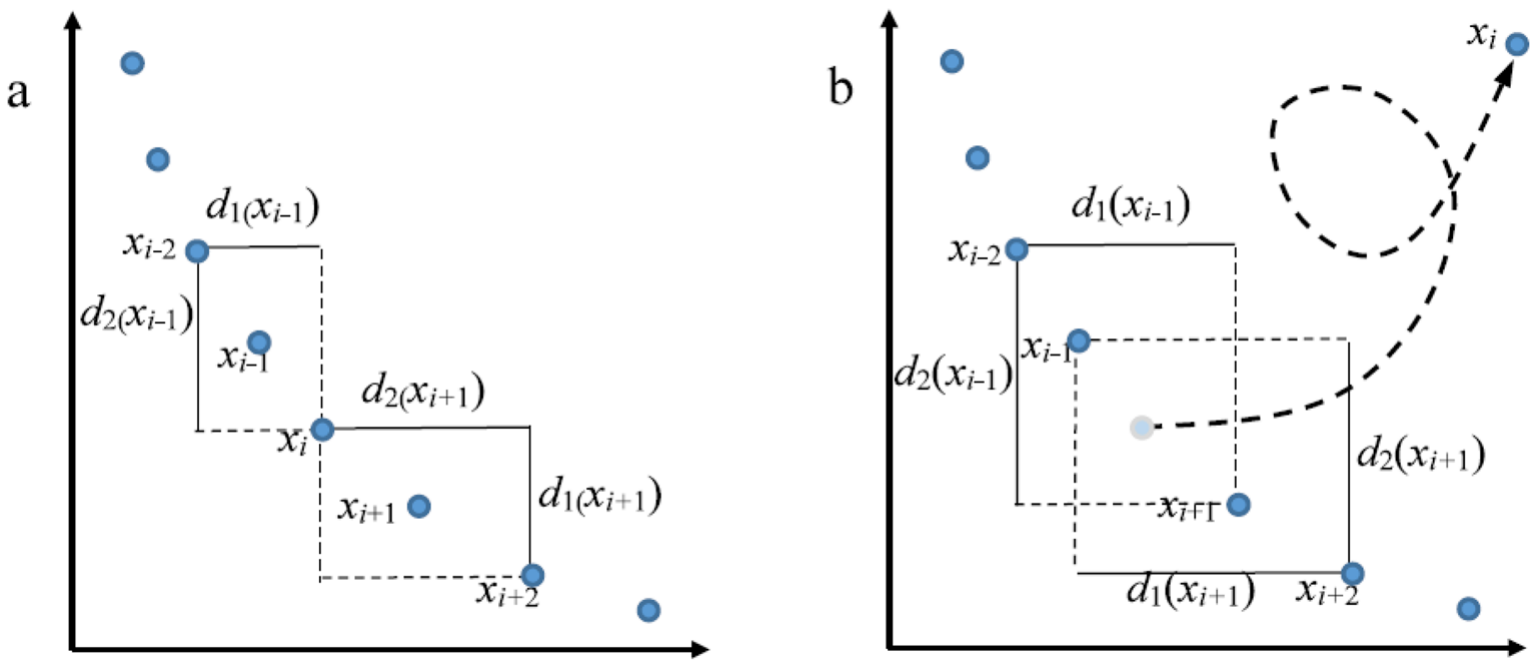
Change of the crowding distance of solutions adjacent to the removed one using DECD for two objectives. (**a**) Crowding distances of $x_{i - 1}$ and $x_{i + 1}$ before $x_{i}$ is removed, and (**b**) crowding distances of $x_{i - 1}$ and $x_{i + 1}$ after $x_{i}$ is removed.

After the *i*th solution with the current smallest crowding distance is removed, the crowding distances of the last and next solutions adjacent to the *i*th solution are changed as follows, respectively:

11 $$\left\{ {\begin{array}{*{20}l} {d_{k} \left( {x_{i - 1} } \right) = \frac{{\left| {f_{k} \left( {x_{i + 1} } \right) - f_{k} \left( {x_{i - 2} } \right)} \right|}}{{\max \left( {f_{k} } \right) - {\text{min}}\left( {f_{k} } \right)}}} \hfill \\ {D\left( {x_{i + 1} } \right) = \mathop \sum \limits_{k = 1}^{m} d_{k} \left( {x_{i - 1} } \right)} \hfill \\ \end{array} } \right.$$

12 $$\left\{ {\begin{array}{*{20}l} {d_{k} \left( {x_{i + 1} } \right) = \frac{{\left| {f_{k} \left( {x_{i - 1} } \right) - f_{k} \left( {x_{i + 2} } \right)} \right|}}{{\max \left( {f_{k} } \right) - {\text{min}}\left( {f_{k} } \right)}}} \hfill \\ {D\left( {x_{i + 1} } \right) = \mathop \sum \limits_{k = 1}^{m} d_{k} \left( {x_{i + 1} } \right)} \hfill \\ \end{array} } \right.$$

where $d_{k} \left( {x_{i - 1} } \right)$ is the side length of the cuboid of the (*i*−1)th solution, and ${\text{D}}\left( {x_{i - 1} } \right)$ is the crowding distance of the (*i*−1)th solution. Figure 6 illustrates the change of the crowding distance of the solutions adjacent to the removed one using DECD for two objectives. When *n* solutions are removed for establishing an external archive using the DECD method, only crowding distances of 2*n* solutions need to be recalculated. Liu and Chen^41^ proposed a crowding distance elimination (CDE) method for improving solution diversity. Luo et al.^42^ devised a dynamic crowding distance-based diversity maintenance strategy (DCDDMS) to enhance solution diversity horizontally. DECD, CDE, and DCD-DMS are similar in that the sorting of the residual solutions is reconsidered whenever a solution with the minimum crowding distance so far is removed. The difference among them is that whenever the solution with the current minimum crowding distance is removed, the crowding distances of residual solutions in the other methods are recalculated. In contrast, only the crowding distances of the immediately adjacent solutions to the removed solution in the DECD method are recalculated. The DECD method reduces the computational complexity of computing the crowding distance of solutions from *O(TMN*2) to *O(TMN)*.

**Figure 7:**
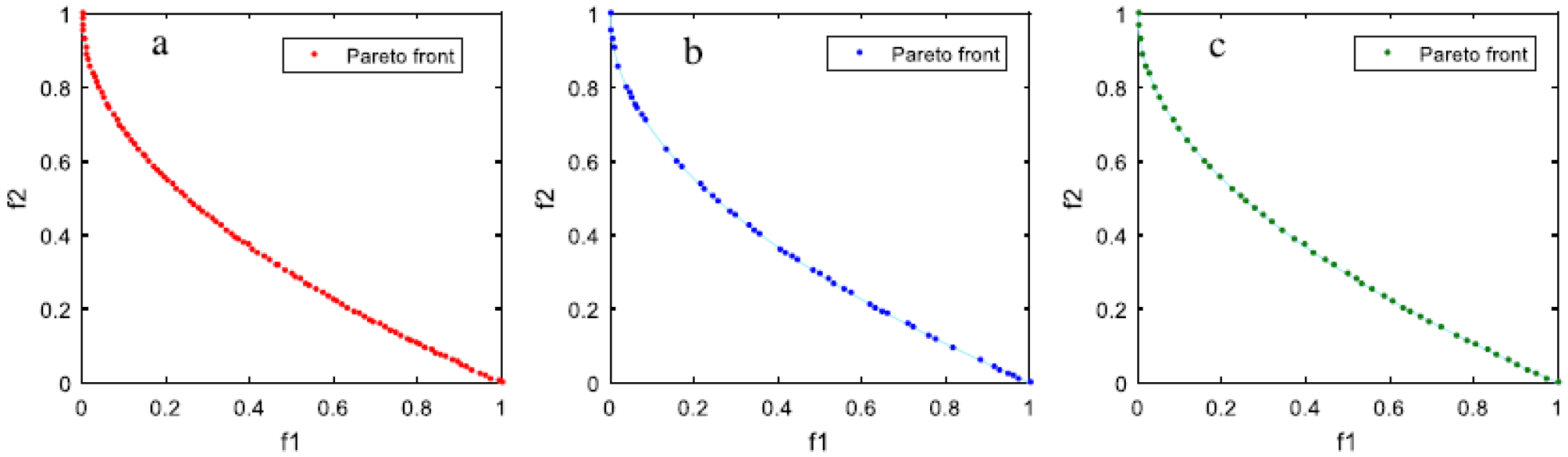
(**a**) Pareto optimal front for two objectives, (**b**) external archive based on crowding distance, and (**c**) external archive based on DECD.

**Figure 8:**
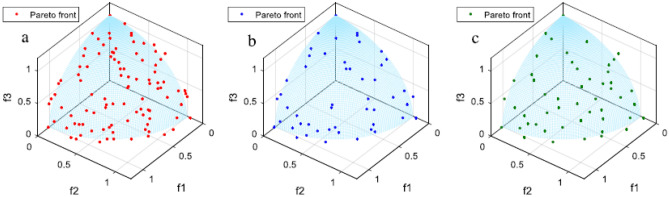
(**a**) Pareto optimal front for three objectives, (**b**) external archive based on crowding distance, and (**c**) external archive based on DECD.

Figure 7 shows external archiving procedures based on crowding distance and DECD for two objectives. There are 100 non-dominated solutions in Fig. 7a, in which 50 must be removed. Figure 7b, c display the external archives based on crowding distance and DECD, respectively. Figure 8 shows external archiving procedures based on crowding distance and DECD for three objectives. There are 100 non-dominated solutions in Fig. 8a, in which 50 must be removed. Figure 8b, c depict external archives based on crowding distance and DECD, respectively. Evidently, the solutions in the external archiving based on the DECD method maintain better diversity and distribution than those in the external archiving based on the crowding distance.

### Guided foraging

In AHA, a hummingbird tends to move to the food source with the highest visit level when performing guided foraging. When there are multiple food sources with the highest visit level, the one with the best fitness is selected as the target food source for foraging. In MOAHA, when there are multiple food sources with the highest visit level, the one in the better front based on NDS is selected as the target food source. If there are still multiple solutions in the same better front, one is randomly selected as the target food source.

After a hummingbird visits the target food source using Eq. (8), the visit level of the target food source for this hummingbird in the visit table is set to 0, and then the visit levels of the other food sources are increased by 1. After performing guided foraging, a new food source (candidate solution) is obtained, and the solution update strategy based on NDS is performed.

If this new food source meets the requirement of update condition in Eq. (9), this hummingbird will abandon the previous food source and station in this new food source (candidate solution). Otherwise, it still rests on the previous food source. This solution update results in the visit level update of the food source for all other hummingbirds. If the solution update succeeds for a hummingbird, the visit level of the updated food source should be set to the highest level of the other food sources plus 1 for every other hummingbird. If the solution update fails for a hummingbird, the visit level does not change.

The following example (a two-objective minimization problem) shows how each hummingbird's target food source is selected and how the visit table is modified when employing the solution update strategy.

**Figure 9:**
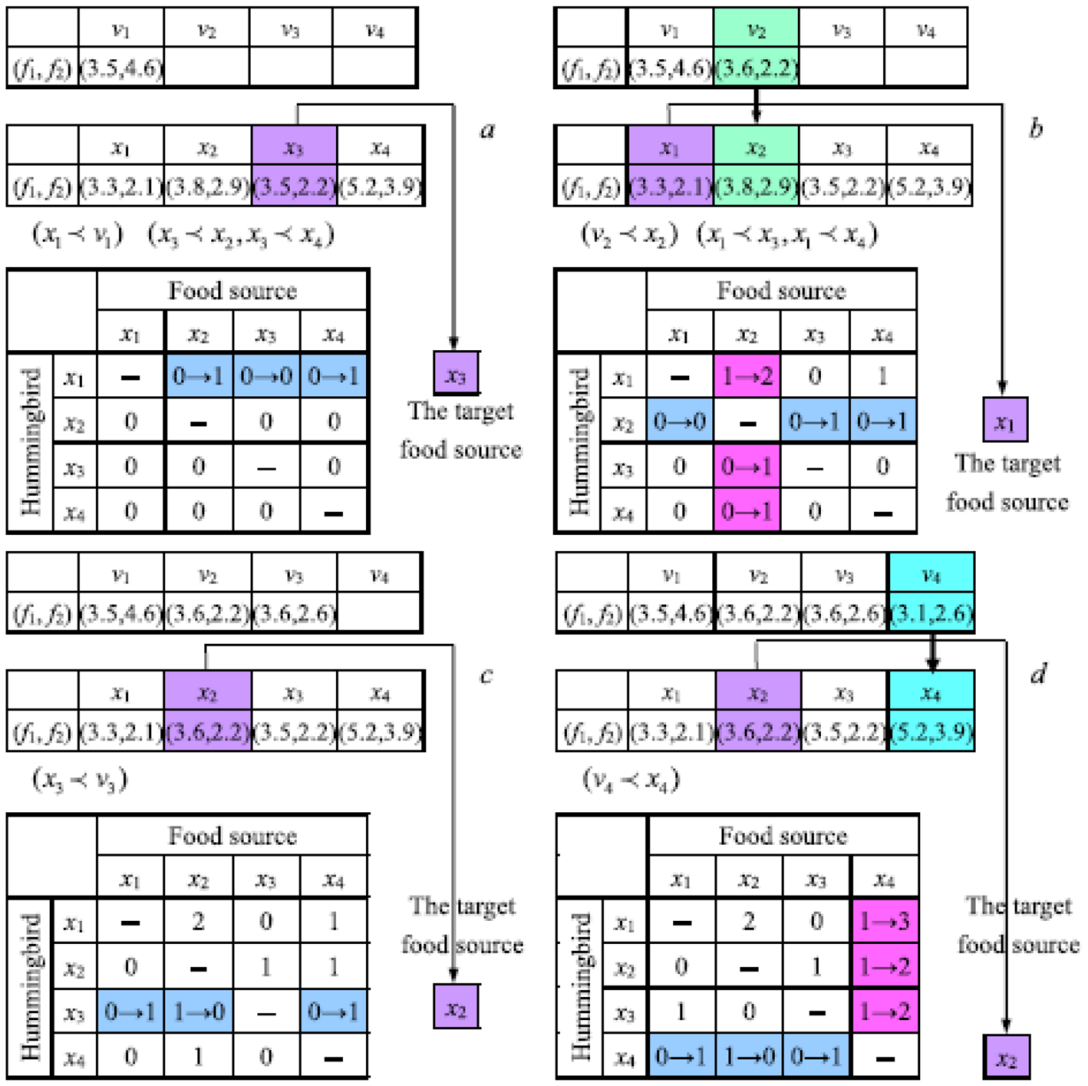
Update of visit table and solutions when performing guided foraging in MOAHA.

In Fig. 9a, assume that four hummingbirds are placed on different food sources, and all visit levels in the visit table are initialized to 0. The first hummingbird finds the food sources of other hummingbirds *x*_2_, *x*_3,_ and *x*_4_ that have the same highest level; since *x*_3_ dominates both *x*_2_ and *x*_4_, the food source of the hummingbird *x*_3_ is treated as the target source of the first hummingbird. After the first hummingbird performs guided foraging via Eq. (8), the visit level of the food source of the hummingbird *x*_3_ is set to 0, and the visit levels of the food sources of hummingbirds *x*_2_ and *x*_4_ are increased by 1. Since the current solution × 1 dominates the candidate solution v_1_, *x*_1_ is not updated by v_1_ (if a solution dominates another solution, the front of this solution is better than the front of the other solution).

In Fig. 9b, the second hummingbird finds the food sources of other hummingbirds *x*_1_, *x*_3_ and *x*_4_ with the same highest level, since *x*_1_ dominates both *x*_3_ and *x*_4_, the food source of hummingbird x_1_ is treated as the target source of the second hummingbird. After the second hummingbird performs guided foraging via Eq. (8), the visit level of the food source of the hummingbird *x*_1_ is set to 0, and visit levels of the food sources of the hummingbirds *x*_2_ and *x*_4_ are increased by 1. Since the candidate solution v_2_ dominates the current solution *x*_2_, *x*_2_ is updated by v_2,_ and the visit level of the source of the hummingbird *x*_2_ for each of the other hummingbirds is modified to the highest visit level increased by 1 in every corresponding row.

In Fig. 9c, the third hummingbird finds the food sources of the hummingbird *x*_2_ that has the highest level, so the food source of the hummingbird *x*_2_ is treated as the target source of the third hummingbird. After the third hummingbird performs guided foraging via Eq. (8), the visit level of the food source of the hummingbird *x*_2_ is set to 0, and visit levels of food sources of the hummingbirds *x*_1_ and *x*_4_ are increased by 1. Since the current solution *x*_3_ dominates the candidate solution v_3_, *x*_3_ is not updated by v_3_.

**Figure 10:**
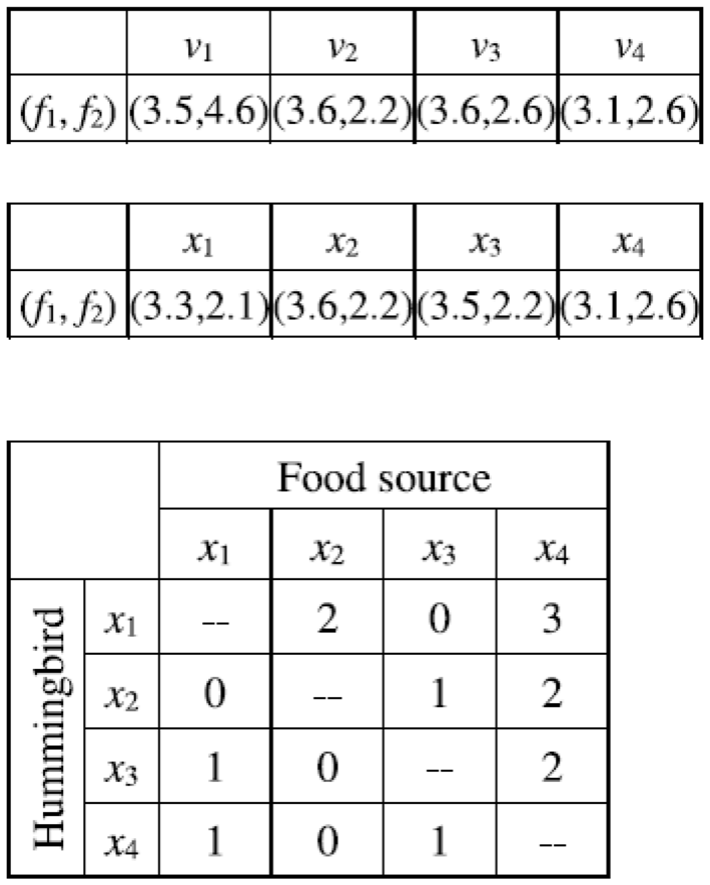
Updated visit table of hummingbirds after one iteration.

In Fig. 9d, the fourth hummingbird finds the food source of the hummingbird × 2 that has the highest visit level, and the food source of the hummingbird × 2 is treated as the target source of the fourth hummingbird. After the fourth hummingbird performs guided foraging via Eq. (8), the visit level of the food source of the hummingbird × 2 is set to 0, and visit levels of the food sources of the hummingbirds × 1 and × 3 are increased by 1. Since candidate solution *v*4 dominates the current solution × 4, × 4 is updated by *v*4, and the visit level of the source of the hummingbird × 4 for each of the other hummingbirds is modified to the highest visit level increased by 1 in every corresponding row. When one iteration is achieved for the four hummingbirds, their updated solutions and visit table are shown in Fig. 10.

### Territorial foraging

When a hummingbird performs a local search in its territory, a new food source is produced as a candidate solution. The mathematic equation simulating the territorial foraging of hummingbirds is given below to improve the solution diversity in MOAHA:

13 $$\left\{ {\begin{array}{*{20}l} {v_{i} \left( {t + 1} \right) = x_{i} \left( t \right) + D \cdot b \cdot x_{i} \left( t \right)rand < 0.5} \hfill \\ {v_{i} \left( {t + 1} \right) = x_{i} \left( t \right) + D \cdot b \cdot x_{a} \left( t \right)rand \ge 0.5} \hfill \\ \end{array} } \right.$$

where *xa*(*t*) is a solution randomly chosen from the external archive.

When a hummingbird performs territorial foraging, the visit levels of the food sources of other hummingbirds are increased by 1. After completing the territorial foraging, a new food source (candidate solution) is found, and the solution update is performed according to Eq. (13). If the solution update succeeds, the visit level of the updated food source is set to the highest level of the other food sources plus 1; if the solution update fails, the visit level does not change. The pseudocode of the territorial foraging strategy of MOAHA is described in Fig. 9, showing the update of the visit table when the first hummingbird performs territorial foraging.

**Figure 11:**
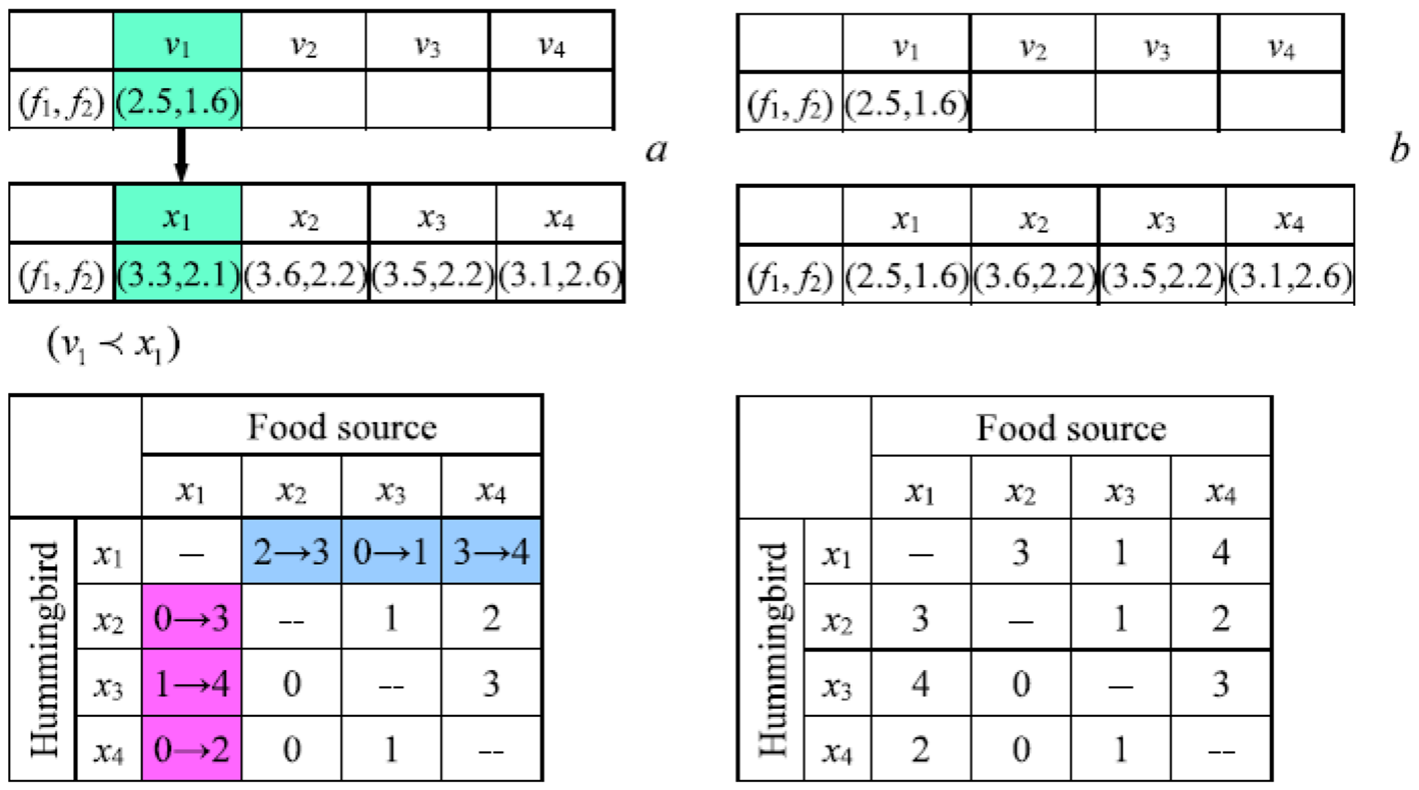
Update of visit table and solutions when performing territorial foraging in MOAHA.

For brevity, Fig. 11 only shows the solution update procedure of the first hummingbird in territorial foraging. Based on Fig. 11a, when the first hummingbird performs territorial foraging in its local region, the visit levels of other hummingbirds × 2, × 3, and × 4 are increased by 1. After performing territorial foraging, since candidate solution *v*1 dominates the previous solution × 1, the previous solution × 1 is replaced by the candidate solution *v*1, and the source of the hummingbird × 1 for each of the other hummingbirds is modified to the highest visit level increased by 1 in every corresponding row. Figure 11b shows the updated solution and visit table after the first hummingbird performs the territorial foraging.

Note that the solution update procedure is the same for both guided foraging and territorial foraging; the difference between them in the updating visit table is that the visit level of the visited food source is initialized to 0 in guided foraging, while this step is not implemented in territorial foraging as a hummingbird visits its own territory instead of the existing food source of other hummingbirds.

### Migration foraging

For AHA, when performing migration foraging, the hummingbird located at the food source with the worst nectar-refilling rate tends to migrate to a new food source produced randomly in the entire search space. The food source with the worst nectar-refilling rate is the solution with the worst fitness in all the solutions. For MOAHA, the food sources with the worst nectar-refilling rate are defined as the solutions in the worst front based on NDS.

The migration foraging of hummingbirds in MOAHA is expressed as follows:

14 $$\left\{ {\begin{array}{*{20}l} {wor \in F_{end} } \hfill \\ {x_{wor} \left( {t + 1} \right) = Low + r \cdot \left( {Up - Low} \right)} \hfill \\ \end{array} } \right.$$

where *Fend* is the worst front, *r* is the rand number in [0, 1], and *Up* and *Low* are the upper and lower boundaries. When migration foraging is completed, the visit table is updated, and the update procedure is similar to those of the other two foraging strategies.

### Multi-objective artificial hummingbird algorithm (MOAHA)

MOAHA begins with a random group of solutions by creating an external archive with a fixed number for a particular multi-objective optimization task and initializing a random population of hummingbirds. After initializing a visit table, all non-dominated solutions from the original population are recorded in the archive. Each time, MOAHA has a 50% chance of choosing between territorial foraging and guided foraging. When a hummingbird participates in guided foraging, it usually shifts its position concerning the target food supply determined by the visit table and dominance relationship. When a hummingbird is territorially foraging, it shifts its position toward the community in its area. The solution update based on NDS is performed in line with Eq. (8) following the completion of one forage, which modifies the visit table.

Migration foraging is used every 2n iterations, and the visit table and worst-front solutions are both randomly initialized in the search field.

**Figure 12:**
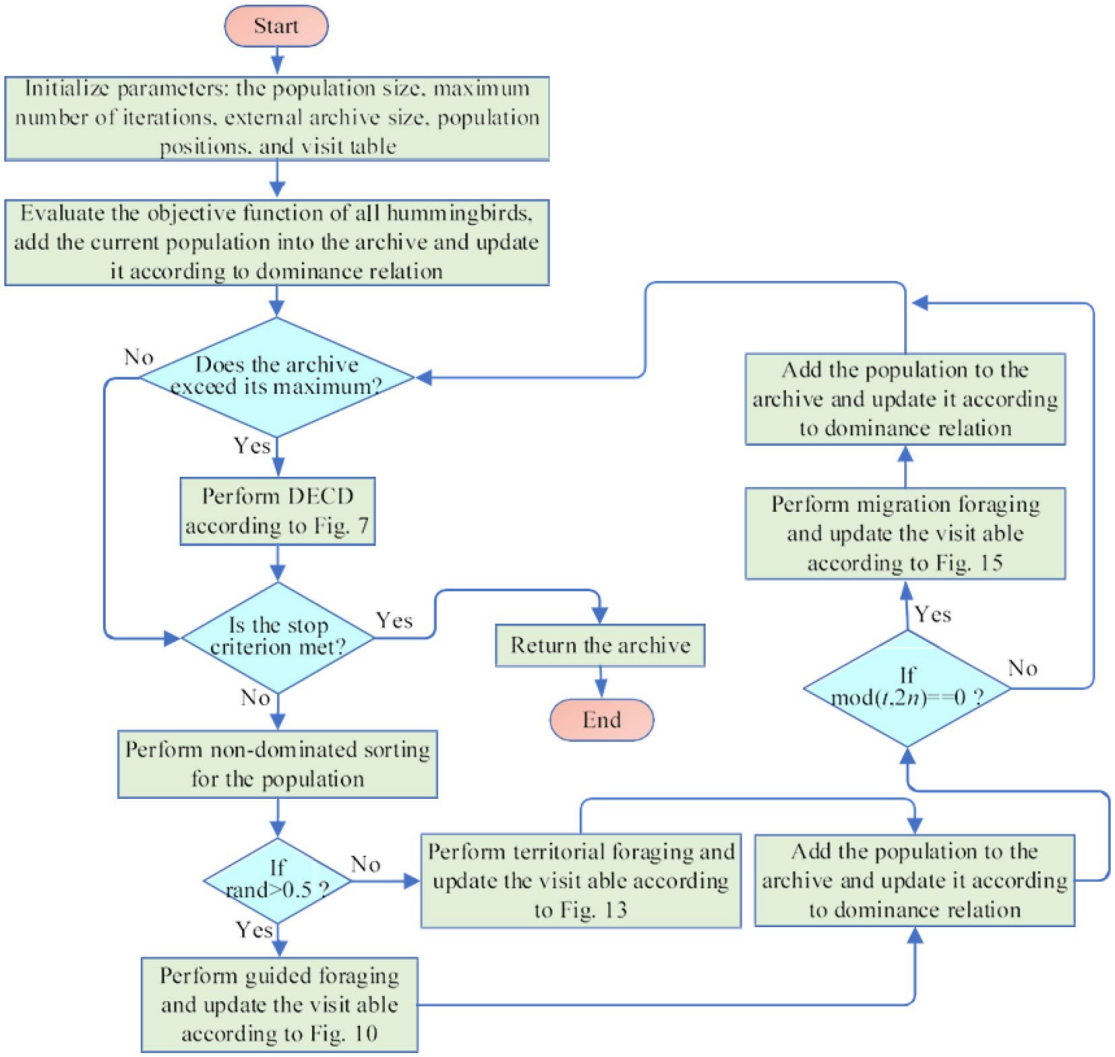
The flowchart of MOAHA.

At the end of each iteration, non-dominated solutions in the new population are added to the archive. The external archiving mechanism based on DECD is triggered if the archive size exceeds the predetermined limit. These processes are repeated until the maximum number of iterations is reached. The PF is eventually returned along with the ideal non-dominated solutions from the archive. The MOAHA diagram is explained in Fig. 12.

This is a theoretical investigation of combining different techniques.

1. MOAHA has an internal external archive with a preset size that can store and recover non-dominated optimal solutions. Archives may be a great resource for all MOAs. Solutions are managed in archives using the DECD technique. The literature has established that the crowing distance method is suitable for maintaining solution variety. Contrarily, the one-step elimination method of crowding distance is useful for maintaining archives. The phase-out technique can greatly improve the distribution of solutions. As a result, the DECD approach can considerably improve the consistency and diversity of the solutions.
2. The NDS approach can divide all the solutions into multiple fronts, allowing for easy comparison of both the existing and potential solutions based on front ranks^9^. Better non-dominated solutions can then be archived and handed on to the following iteration to aid in the search operations of the other users. Therefore, a solution updating method with NDS can improve the algorithm's ability to converge to optimal solutions.
3. Since MOAHA does a global search using the same methods as AHA, convergence may result. The performance of searches in terms of exploration, exploitation, and convergence has been theoretically assessed for these procedures, three foraging methods, and a visit table^40^. Because MOAHA receives all the advantages of AHA, searchers can perform both exploitation and exploration in the same manner. The main difference between the two is that while AHA simply stores one optimal solution and later fine-tunes it, MOAHA looks through various non-dominated solutions and saves them to the archives.

### Other multi-objective algorithms

#### Multi-objective moth swarm algorithm (MOMSA)

Single-objective MSA, proposed by Mohamed et al.^43^, is inspired by the behavior of moths in nature. Moths try to hide from predators during the day while looking for food resources at night with a celestial navigation technique. They fly in a straight line over a long distance by steering their locomotion in a steady angle relative to moonlight as the celestial far-distant point of light. In the MSA, the possible solution is represented by the position of the light source, and the quality of this solution is considered the luminescence intensity of the light source. Three groups of moths (pathfinders, prospectors, and onlookers) are considered in the MSA. Pathfinders can find the best position over the optimization space with the First-In, Last-Out principle to guide the movement of the main swarm. Prospectors tend to wander into a random spiral path near the light sources marked by the pathfinders. Onlookers drift directly toward the best global solution (moonlight) achieved by the prospectors^43^.

The dominant features of the algorithm (such as moonlight and pathfinder moth) must be properly defined to convert MSA into an efficient multi-objective optimization algorithm. In normal optimization problems for MSA, only one objective function should be minimized or maximized. In this condition, three types of moths, including pathfinder, prospector, and onlooker, follow the process of searching and reaching the final objective in three stages of cognition, transverse orientation, and constellation orientation. The prospector moths randomly move in a maze toward the best pathfinder moths (responsible for guiding the prospectors). Following this process, the onlooker moths move directly toward the best global optimal solution (moonlight) detected by the prospectors. The extraordinary performance of the MSA algorithm in solving single-objective problems, proven in various studies, has turned it into one of the most powerful metaheuristic algorithms^44,45^. A multi-objective moth swarm algorithm (MOMSA) with the capability of solving complex and large-scale problems was proposed by Sharifi et al.^44^ to solve multi-objective constrained problems. Since more than one function must be maximized in MOPs, the definition for selecting the type of moths and the best value (moonlight) is changed in MSA to the multi-objective space. The crowding-distance mechanism selects the most efficient (best) solutions in the population as the pathfinder moths and moonlight (global optimum). Sharifi et al.^44^ presented a full description of the method and key components of the MOMSA algorithm.

#### Multi-objective material generation algorithm (MOMGA)

The MGA algorithm, a metaheuristic algorithm recently developed by Talatahari et al.^46^, considers the general aspects of material generation in nature, along with the basic and advanced principles of chemistry, including chemical compounds and chemical reactions^46^.

The multi-objective version of MGA for solving multi-objective optimization problems was presented by Nouhi et al.^47^. MOMGA has three new mechanisms for solving multi-objective optimization.

The first mechanism introduced into MGA is the archive, which serves as a storage facility for storing or restoring the derived Pareto optimal solutions. The archive has a single controller that manages which solutions are added to the archive and when the archive is full. There is a limit to the number of solutions that can be stored in the archive. The residents of the archive are compared to the non-dominated solutions created. Three major scenarios are possible:

I. If at least one member in the archive dominates the new solution, it is not allowed to enter the archive.
II. The new solution may be added to the archive if it dominates at least one solution in the archive by omitting the one already in the archive.
III. If the new and archive solutions do not dominate each other, the new solution is added to the archive.

The grid mechanism included in MGA is the second effective technique for enhancing non-dominated solutions in the archive. If the archive is full, the grid technique will be utilized to reorganize the object space's segmentation and find the most populated area to eliminate a solution. The additional member should be included in the least crowded segment to boost the variety of the final approximated Pareto optimal front. As the number of possible solutions in the hypercube increases, the possibility of deleting a solution rises. If the archive is full, the most crowded areas are chosen first, and a solution from one of them is randomly deleted to make way for the new solution. When a solution is placed outside the hypercubes, a special case arises. All segments in this scenario have been expanded to fit the most recent solutions. As a result, the segments of alternative solutions can also be changed.

Due to Pareto optimality, solutions in a multi-objective search space cannot be compared; hence, the leader selection mechanism is the last machine in MGA. Consequently, MGA includes a leader selection method to address this problem.

The search leaders guide the other search candidates to possible areas of the search space to attain a solution close to the global optimum. As previously stated, the archive contains only the best non-dominated solutions. The leader selection mechanism chooses the least crowded portions of the search space and presents the best as non-dominated answers.

A full description of the MOMGA algorithm is provided by Nouhi et al.^47^.

#### Performance criteria for MOPs

GD^48^, S^49^, Δ^50^, and MS^36^ are the four performance parameters used in this study to assess the performance of the exploitation algorithms.

15 $$GD = \frac{1}{NPF}\left( {\mathop \sum \limits_{i = 1}^{NPF} d_{i}^{2} } \right)^{\frac{1}{2}}$$

where *d* is the Euclidean distance between the i-th member in *PF*_*g*_ and the closest member in *PF*_*optimal*_, where *NPF* is the total number of members discovered in the Pareto front. The best *GD* criterion that can be obtained is zero, which indicates that *PF*_*g*_ is precisely on the optimal line or *PF*_*optimal*_.

16 $$S = \sqrt {\frac{1}{NPF - 1}\mathop \sum \limits_{i = 1}^{NPF} (d_{i} - \overline{d})^{2} }$$

In Eq. (16), $d_{i} = min_{j} (\left| {f_{1}^{i} \left( x \right) - f_{1}^{j} \left( x \right)} \right| + \left| {f_{2}^{i} \left( x \right) - f_{2}^{j} \left( x \right)} \right|$, *i*,*j* = 1,2,…,*NPF* and $\overline{d}$ is the average of all *d*_*i*_. The lowest value of *S* in *PF*_*g*_ leads to the best uniform distribution. As a result, if all non-dominated solutions are distributed equally throughout *PF*_*g*_, the value of *S* is zero.

17 $$\Delta = \frac{{d_{f} + d_{l} + \mathop \sum \nolimits_{i = 1}^{NPF} \left| {d_{i} - \overline{d}} \right|}}{{d_{f} + d_{l} + \left( {NPF - 1} \right)\overline{d}}}$$

where in *PF*_*optimal*_ and *PF*_*g*_, df and dl represent the separation between extremum solutions (beginning and finishing points). The distance di separate any point in *PFg* and the closest point in PFotpimal.

This metric represents a graph's point-to-point distance. The value of *Δ* is always greater than zero, and a lower value indicates a better distribution and spread of solutions.

When *Δ* is zero, it is a perfect condition showing that $d_{i} = \overline{d}$ for all non-dominant points.

18 $$MS = \left[ {\frac{1}{m}\mathop \sum \limits_{i = 1}^{m} \left[ {\frac{{\min \left( {f_{i}^{max} ,F_{i}^{max} } \right) - {\text{max}}\left( {f_{i}^{min} ,F_{i}^{min} } \right)}}{{F_{i}^{max} - F_{i}^{min} }}} \right]^{2} } \right]^{\frac{1}{2}}$$

where $f_{i}^{max}$ and $f_{i}^{min}$ are, respectively, the maximum and minimum values of the ith objective in *PF*_*g*_, and $F_{i}^{max}$ and $F_{i}^{min}$ are the maximum and minimum of the ith target in *PF*_*optimal*_, respectively. A higher MS score indicates a wider range of solutions.

Generally, the performance criteria of multi-objective problems are used to evaluate the performance of multi-objective algorithms. The closer the GD, S, and Δ criteria to zero and the higher the MS criterion, the greater the superiority and performance of the multi-objective algorithm.

### Long-term multi reservoir-multi objective simulation–optimization model

The optimal monthly release rates from the dams' reservoirs serve as the decision factors in the multi-reservoir, multi-objective optimization model. In succession, these release values in the system of five Karun dam reservoirs include releases intended to satisfy downstream demand, control flooding, and provide electric energy. The MOAHA algorithm has 1800 decision variables in the system because the planning horizon for this study is 180 months (from 2000 to 2015). Reservoir downstream needs and releases from energy outputs are the decision variables, while storage volume and input to reservoirs are the state variables for each time. The volume of the river, the height of evaporation, the height of precipitation, and the volume of requirements every month are the input data for the model.

For deterministic optimization of the reservoir system in reservoir problems, the usual objective function is as follows:

19 $$Min\left( {Max} \right) Z = \mathop \sum \limits_{t = 1}^{T} f\left( {S_{t} ,Re_{t} } \right)$$

In this context, *t* stands for the intended period index, *T* for the number of exploitation periods, *Z* for the target that should be minimized or maximized, $Re_{t}$ is the release, and $S_{t}$ is the storage in period *t*. As a fundamental relationship in the state-space equations, the conservation of mass in the system is as follows:

20 $$S_{t + 1} = S_{t} + Q_{t} - Re_{t} - Sp_{t} - Loss_{t}$$

Thus, $Q_{t}$ is the inflow during period* t*, $Sp_{t}$ is the overflow during period *t*, and $Loss_{t}$ is the loss during period *t*. The upper and lower storage volume limitations should be set to meet the needs of renewable flood control volume objectives and ensure a minimum level of balance for reservoir dead volume and power plant operation.

21 $$S_{Min} \le S_{t} \le S_{Max}$$

22 $$Re_{Min} \le Re_{t} \le Re_{Max}$$

In these equations, $S_{Min}$ and $S_{Max}$ are the minimum and maximum storage volumes,$Re_{Min}$ and $Re_{Max}$ are the minimum and maximum release volumes, respectively.

Evaporation is used to evaluate reservoir losses while accounting for the non-linear relationship between reservoir surface and volume based on Eqs. (23) to (25):

23 $$Loss_{t} = Ev_{t} \times \overline{{A_{t} }} /1000$$

24 $$\overline{{A_{t} }} = \left( {A_{t} + A_{t + 1} } \right)/2$$

25 $$A_{t} = a \times S_{t}^{2} + b \times S_{t} + c$$

In these equations, *a*, *b*, and *c* are constant coefficients for converting reservoir volume to the corresponding level of the reservoir, respectively, $Ev_{t}$ the average drop during period *t* (evaporation minus precipitation) in millimeters, $\overline{{A_{t} }}$ the average level of reservoir during period *t* in square kilometers, and $A_{t}$ and $A_{t + 1}$ the amount of level in reservoir at beginning and end of period *t*.

The objectives of providing downstream needs, flood control, and electric energy production are considered for the optimal use of the reservoir system. Three outlets—an electrical outlet, a downstream demand supply outlet, and a flood control outlet—are considered for the reservoir system. In various exploitation modes, water is released first from the electrical outlet, then from the supply outlet, and finally, from the flood control outlet.

In this way, the other two outlets are not used until the electricity outlet is used to its maximum capacity, and after using the demand–supply outlet to its maximum capacity, the flood control outlet is utilized. $Re_{Max}^{Power}$ is the maximum output capacity for power production, $Re_{Max}^{De}$ is the maximum output capacity for demand supply, and $Re_{Max}^{FC}$ is the maximum output capacity for flood control, all measured in million cubic meters. Note that in this instance, $Re_{t}$ is between zero and the total of the maximum output of reservoir, and $Re_{t}^{Power}$, $Re_{t}^{De}$ and $Re_{t}^{FC}$ respectively denote the quantity of release from electric output, demand supply, and flood control measured in a million cubic meters for each period t.

### Operating the reservoir system for downstream demands supply

In this case, the goal is to minimize the sum of squared difference from the requirement in period t, displayed as the following equation:

26 $$Min f_{1} = \mathop \sum \limits_{t = 1}^{T} \left( {\frac{{Re_{t}^{Power} + Re_{t}^{De} - De_{t} }}{{De_{Max} }}} \right)^{2}$$

Here, $f_{1}$ is the sum of the squared difference of monthly release ($Re_{t}^{Power} + Re_{t}^{De}$) from requirement in period t, $De_{Max}$ is the maximum monthly requirement of the reservoir, and $De_{t}$ is the amount of requirement.

### Operating the reservoir system for flood control

Here, all the equations are like the exploitation for satisfying downstream needs, and only the objective function is as follows:

27 $$Min f_{2} = \mathop \sum \limits_{t = 1}^{T} \left( {\frac{{S_{t} - S_{t}^{target} }}{{S_{Max} }}} \right)^{2}$$

Here, $S_{t}^{target}$ is the desired flood control volume in t period per million cubic meters. In the introduced objective function, the objective is to keep a constant volume around $S_{t}^{target}$ in all the exploitation periods. In fact, if the reservoir volume is more than the desired volume, the objective is flood control; if it is less than the desired volume, the objective of supplying downstream needs will not be met and will cause deviation from $S_{t}^{target}$ in each period.

### Operating the reservoir system for hydropower energy generation

One must consider the problem's complexity in terms of constraints and nonlinear conditions to make the best use of electricity. For this purpose, the final objective function, including minimizing the lack of production power compared to the installed capacity of power plants, was used according to Eq. (28).

28 $$Min F = \mathop \sum \limits_{i = 1}^{N} \mathop \sum \limits_{t = 1}^{T} \left( {1 - \frac{{P_{i,t} }}{{PPC_{i} }}} \right)$$

Here, $P_{i,t}$ is the production energy of reservoir i in period t per MW, and $PPC_{i} { }$ is the installation capacity of reservoir i per MW. According to Eqs. (29) to (40), additional constraints and criteria were applied to optimize hydropower production:

29 $$P_{i,t} = g \times e_{i,t} \times \frac{{RP_{i,t} }}{{PF_{i} }}/Mul_{t} \times \left( {\overline{{H_{i,t} }} - TW_{i,t} } \right)/1000$$

30 $$\overline{{H_{i,t} }} = \left( {H_{i,t} + H_{i,t + 1} } \right)/2$$

31 $$H_{i,t} = a_{0i} + a_{1i} \cdot S_{i,t} + a_{2i} \cdot S_{i,t}^{2} + a_{3i} \cdot S_{i,t}^{3} + a_{4i} \cdot S_{i,t}^{4}$$

32 $$TW_{i,t} = b_{0i} + b_{1i} \cdot Re_{i,t}^{Power} + b_{2i} \cdot \left( {Re_{i,t}^{Power} } \right)^{2} + b_{3i} \cdot \left( {Re_{i,t}^{Power} } \right)^{3} + b_{4i} \cdot \left( {Re_{i,t}^{Power} } \right)^{4}$$

33 $$RPS_{i,t} = Re_{i,t}^{Power} - RP_{i,t}$$

34 $$0 \le P_{i,t} \le PPC_{i}$$

35 $$S_{i,t + 1} = S_{i,t} + Q_{i,t} + Re_{i - 1,t}^{Power} - Re_{i,t}^{Power} - Sp_{i,t} - Loss_{i,t}$$

36 $$Loss_{i,t} = Ev_{i,t} \times \overline{{A_{i,t} }} /1000$$

37 $$\overline{{A_{i,t} }} = \left( {A_{i,t} + A_{i,t + 1} } \right)/2$$

38 $$A_{i,t} = c_{0i} + c_{1i} \cdot S_{i,t} + c_{2i} \cdot S_{i,t}^{2} + c_{3i} \cdot S_{i,t}^{3} + c_{4i} \cdot S_{i,t}^{4}$$

39 $$S_{Min\;i} \le S_{i,t} \le S_{Max\;i}$$

40 $$Re_{Min\;i} \le Re_{i,t} \le Re_{Max\;i}$$

where ${\text{e}}_{{{\text{i}},{\text{t}}}}$ is the reservoir power plant’s efficiency, assumed to be constant over all periods, and g is the acceleration of the earth’s gravity, which is equal to 9.81 m per square second. ${\text{PF}}_{{\text{i}}}$ is the reservoir power plant’s operating coefficient, and ${\text{Mul}}_{{\text{t}}}$ is the ratio of million cubic meters to cubic meters per second over the course of a time interval of t. ${\text{H}}_{{{\text{i}},{\text{t}}}}$ represents the average water level of reservoir i for t meters, ${\text{H}}_{{{\text{i}},{\text{t}} + 1}}$ represents the water level of reservoir i at the start of period t (meters), $TW_{i,t}$ represents the water level of reservoir i at the end of period t (meters), $a_{0i}$, $a_{1i}$, $a_{2i}$, $a_{3i}$ and $a_{4i}$ indicate the constant coefficients used to convert reservoir volume to the corresponding height in reservoir i, constant coefficients of conversion ${\text{b}}_{{0{\text{i}}}}$, ${\text{b}}_{{1{\text{i}}}}$, ${\text{b}}_{{2{\text{i}}}}$, ${\text{b}}_{{3{\text{i}}}}$ and ${\text{b}}_{{4{\text{i}}}}$ output water from the power plant to the elevation of the reservoir i, ${\text{RPS}}_{{{\text{i}},{\text{t}}}}$ dentoes the volume of water that overflows from reservoir i's hydropower outlet during the t period, and ${\text{RP}}_{{{\text{i}},{\text{t}}}}$ shows the volume of water released from reservoir i's hydroelectric outlet during period t to generate electricity. Reservoir i's natural river influx during period t is represented by ${\text{Q}}_{{{\text{i}},{\text{t}}}}$, its overflow is denoted by ${\text{Sp}}_{{{\text{i}},{\text{t}}}}$, and its overall volume loss over time is represented by ${\text{Loss}}_{{{\text{i}},{\text{t}}}}$. ${\text{A}}_{{{\text{i}},{\text{t}}}}$ and ${\text{A}}_{{{\text{i}},{\text{t}} + 1}}$, which shows the reservoir i's water level at the start and end of t period, respectively, represents the reservoir i's water level in square kilometers. ${\text{Ev}}_{{{\text{i}},{\text{t}}}}$ represents the amount of reservoir i's water lost in millimeters in period t (evaporation minus precipitation). ${\text{S}}_{{{\text{Min}}\;{\text{i}}}}$ is the minimum storage volume of the reservoir i, ${\text{S}}_{{{\text{Max}}\;{\text{i}}}}$ is the maximum storage volume of the reservoir i and ${\text{Re}}_{{{\text{Min}}\;{\text{i}}}}$ and ${\text{Re}}_{{{\text{Max}}\;{\text{i}}}}$ are the minimum and maximum release volumes of the reservoir i, respectively. The constant coefficients of conversion for the reservoir volume to the corresponding level of the reservoir are ${\text{c}}_{{0{\text{i}}}}$, ${\text{c}}_{{1{\text{i}}}}$, ${\text{c}}_{{2{\text{i}}}}$, ${\text{c}}_{{3{\text{i}}}}$ and ${\text{c}}_{{4{\text{i}}}}$. The reservoir i's lowest and maximum release volumes are denoted by ${\text{Re}}_{{{\text{Min}}\;{\text{i}}}}$ and ${\text{Re}}_{{{\text{Max}}\;{\text{i}}}}$, respectively. ${\text{Re}}_{{{\text{i}},{\text{t}}}}^{{{\text{Power}}}}$ is a function of ${\text{Re}}_{{{\text{i}} - 1,{\text{t}}}}^{{{\text{Power}}}}$. The power and amount of hydroelectric energy the reservoir discharged over time t. Power is the amount of water released from a reservoir's hydroelectric output over a given time span t.

### Performance metrics for reservoirs

The final and most crucial phase in employing optimization and simulation models to exploit reservoirs is the evaluation of operation policies.

#### Reliability

Reliability can be defined in two ways: time and volume. Time reliability means the percentage of periods when the system fully supplies the existing needs and does not fail. The following equation is used to determine this parameter’s value.

41 $${\text{Rel}} = \left( {1 - \frac{{{\text{NDe}}_{{\text{f}}} }}{{\text{T}}}} \right) \times 100,\;{\text{ND}}_{{{\text{ef}}}} = number{ }\left( {{\text{De}}_{{\text{i}}} > {\text{Re}}_{{\text{i}}} } \right)$$

where *NDe*_*f*_ stands for the overall number of failures experienced during the operation period, Dei for the demand value experienced during the i period, *Re*_*i*_ for the output value experienced during the i period, and *Rel* for the system's reliability experienced during the i period. The higher the value of this parameter, the higher the time reliability of the system^51^.

#### Vulnerability

This indicator measures the intensity of system failures and is derived from the equation below^51^:

42 $${\text{Val}} = {\text{max}}\left\{ {\frac{{\left( {{\text{De}}_{{\text{i}}} - {\text{Re}}_{{\text{i}}} } \right)}}{{{\text{De}}_{{\text{i}}} }}} \right\} \times 100,\quad {\text{i}} = 1,2, \ldots ,{\text{t}}$$

where Val is the system’s vulnerability, De_i_ is the amount of demand in period i, Re_i_ is the output value in period I, and t is the total number of exploitation periods.

#### Resiliency

This index demonstrates the system’s capacity to alter the state of affairs. Reversibility is the likelihood that a system will resume its desired condition after failure^51^. The equation below can be used to calculate this parameter’s value.

43 $${\text{Res}} = \frac{{\begin{array}{*{20}c} {\text{T}} \\ {\text{N}} \\ {{\text{i}} = 1} \\ \end{array} \left( {{\text{Def}}_{{{\text{i}} + 1}} = 0{ }\left| {{\text{ Def}}_{{\text{i}}} } \right\rangle 0} \right)}}{{\begin{array}{*{20}c} {\text{T}} \\ {\text{N}} \\ {{\text{i}} = 1} \\ \end{array} ({\text{Def}}_{{\text{i}}} > 0)}} \times 100\quad {\text{i}} = 1,2, \ldots ,{\text{T}}$$

where Def_i_ is the deficiency in the i-th period, and $$\begin{array}{*{20}c} T \\ N \\ {i = 1} \\ \end{array} ()$$ is the number of times the condition in parentheses happened.

#### Sustainability index

This index aggregates system performance requirements into a general index based on the following reservoir performance indicators, and aims to enable comparison and decision-making between various scenarios^52^.

44 $${\text{SI}} = \left\{ {{\text{Rel}} \times {\text{Res}} \times \left( {1 - {\text{Vul}}} \right)} \right\}^{1/3}$$

Reliability, resiliency, vulnerability, and sustainability criteria are used to compare different algorithms in reservoir operation problems. The closer the reliability, resiliency, and sustainability indicators to 100% and the closer the vulnerability index to 0%, the greater the success in the investigated problem.

### Multi-objective criteria problems

#### Problem 1 of the Schaffer benchmark

A benchmark problem was provided by Schaffer^53^ to assess the effectiveness of multi-objective optimization techniques. This univariate convex problem has two objectives that must be minimized (Eq. 45). This test problem has served as a reliable benchmark. Almost every multi-objective EA has been tested on this problem.

45 $$\begin{aligned} & Minimize\quad f\left( x \right) = \left( {f_{1} \left( x \right),f_{2} \left( x \right)} \right) \\ & Subject\;to\quad f_{1} \left( x \right) = x^{2} \\ & f_{2} \left( x \right) = \left( {x - 2} \right)^{2} \\ \end{aligned}$$

**Figure 13:**
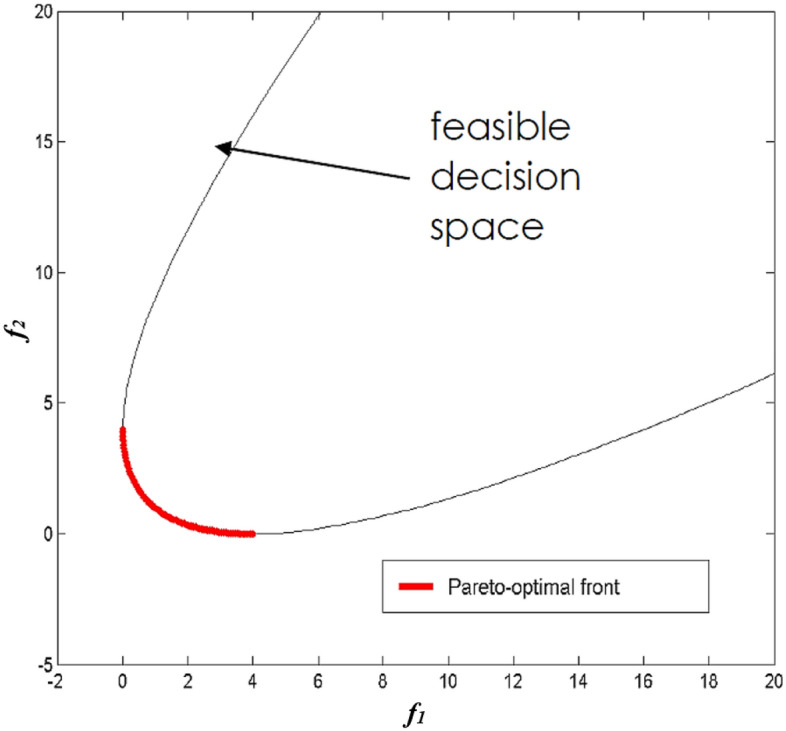
The Schaffer problem’s ideal Pareto front.

This criterion seeks to minimize f1 and f2 simultaneously. The Pareto optimum sites are located in the region where x ∈ [0, 2], as shown in Fig. 13. There is a trade-off between two functions that grow and decrease within this interval because f_1_ and f_2_ increase outside of it.

#### Problem 2 of the MMF1 benchmark

A benchmark concern for CEC 2020 is MMF1. The most recent and official multimodal-multiobjective benchmark set is the CEC 2020. The CEC 2020 benchmark set includes four distinct kinds of benchmark problems: convex linear, convex nonlinear, concave, and concave linear. The shapes of the objective functions and the spaces of the choice variables distinguish these benchmark problems.

The Pareto front (PF) and Pareto optimum set (PS) equations and forms for MMF1 are as follows:

46 $$\left\{ {\begin{array}{*{20}l} {f_{1} = \left| {x_{1} - 2} \right|} \hfill \\ {f_{2} = 1 - \sqrt {\left| {x_{1} - 2} \right|} + 2(x_{2} - \sin (6\pi \left| {x_{1} - 2} \right| + \pi ))^{2} } \hfill \\ \end{array} } \right.$$

where the search space is x_1_ ∈ [− 1, 1] and x_2_ ∈ [1, 3].

**Figure 14:**
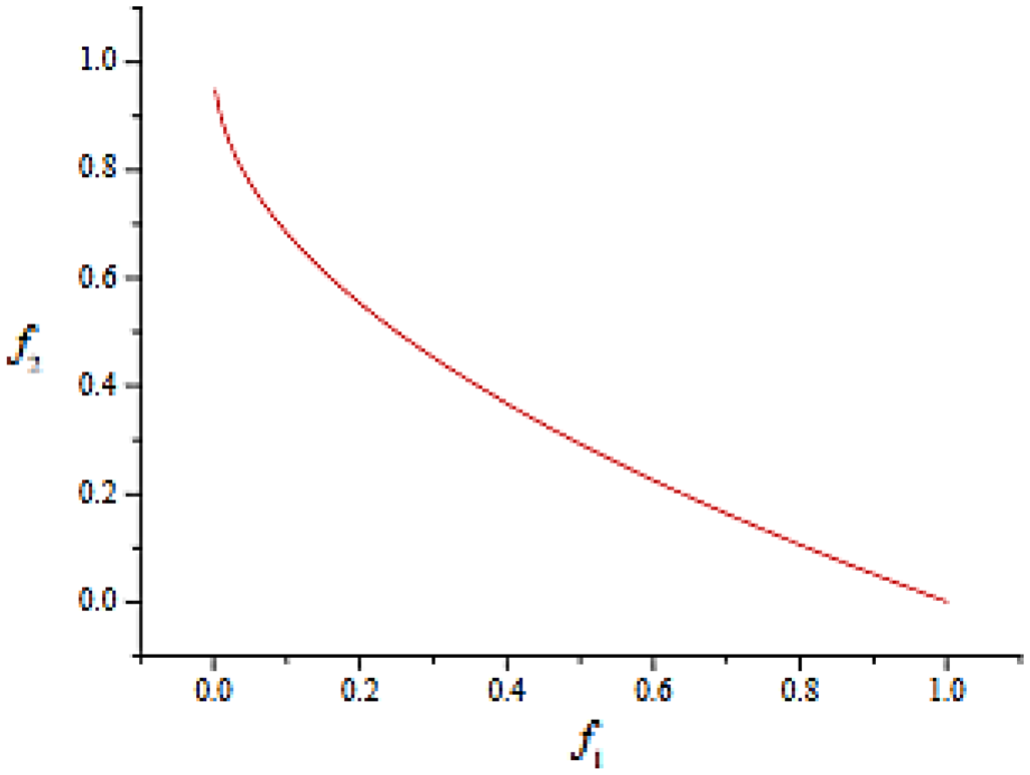
The actual PSs and PF of MMF1.

Pareto global sets (PSs) are:

47 $$\left\{ {\begin{array}{*{20}l} {x_{1} = x_{1} } \hfill \\ {x_{2} = \sin (6\pi \left| {x_{1} - 2} \right| + \pi )} \hfill \\ \end{array} } \right.$$

where $1 \le x_{1} \le 3$ , and the global Pareto Front (PF) is:

48 $$f_{2} = 1 - \sqrt {f_{1} }$$

where $0 \le f_{1} \le 1$. Its actual PS and PF are shown in Fig. 14.

Liang et al.^54^ provide more details on the additional characteristics of the CEC benchmark set problems. The MOAHA multi-objective algorithm's performance in this work was tested and verified using the two benchmark challenges described above.

## Results and discussion

### Multi-objective criteria problems

The MOAHA, MOMSA, and MOMGA algorithms, programmed in MATLAB, had to be assessed for performance and capability. For this purpose, two well-known multi-objective standard benchmark functions were utilized.

The effectiveness of the utilized algorithms was assessed using four widely used evaluation criteria: GD, S, Δ, and MS. The results of the multi-objective benchmark functions Schaffer and MMF1’s standard multi-objective evaluation criteria are illustrated in Table 3.

**Table 3:** The outcomes of the multi-objective algorithm's evaluation parameters in standard benchmark multi-objective functions.

	Benchmark problems	GD	S	Δ	MS
MOAHA	Schaffer	0.00095	0.58155	0.20375	4.00020
	MMF1	0.00833	0.07660	0.17832	1.00000
MOMSA	Schaffer	0.00097	0.67311	0.77148	3.98541
	MMF1	0.00648	0.08074	0.39362	0.99089
MOMGA	Schaffer	0.00114	0.56104	0.71763	4.00245
	MMF1	0.03097	0.07813	0.86098	0.92085

Table 3 shows that all three algorithms performed well in two standard benchmark functions under investigation. The MOAHA algorithm performed better than the other two algorithms with the performance criteria of GD = 0.00095, S = 0.58155, Δ = 0.20375, and MS = 4.00020 in the Schaffer problem and GD = 0.00095, S = 0.58155, Δ = 0.20375, and MS = 4.00020 in the MMF1 problem. The MOAHA algorithm located non-dominated solutions with the shortest distance from the PFoptimal in two benchmark functions under investigation and had a suitable distribution. Besides, the MOAHA algorithm converged well with the PFoptimal (GD criterion).

**Figure 15:**
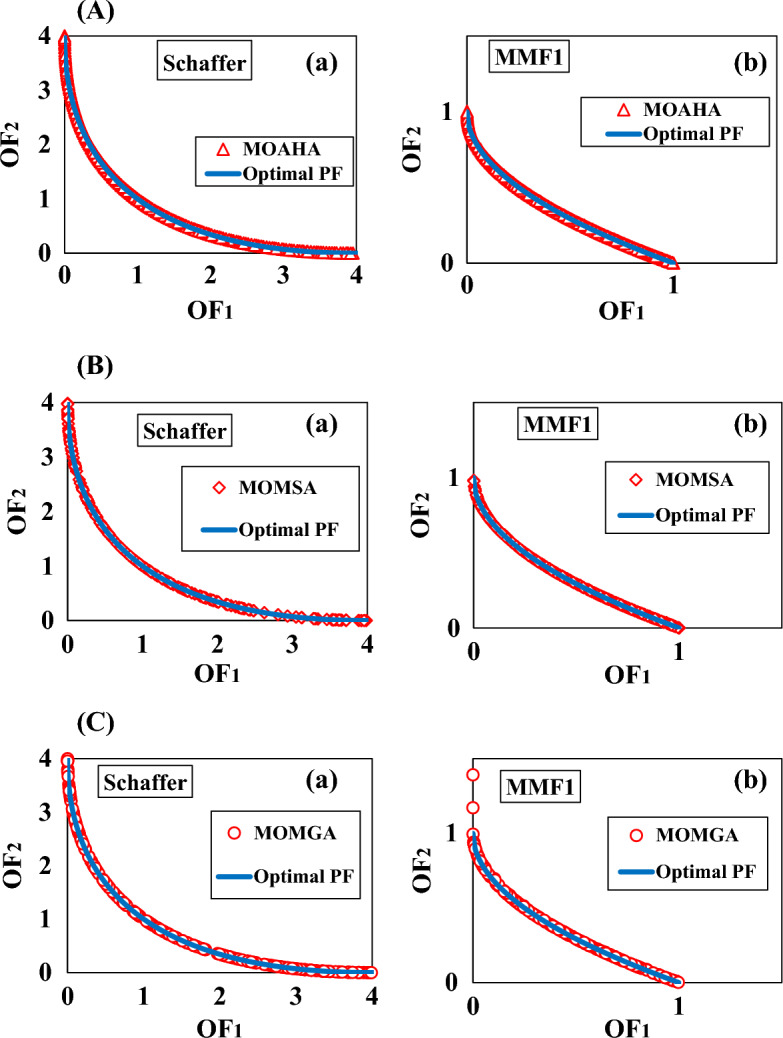
(**a**) The Pareto front resulting from the MOAHA algorithm in multi-objective benchmark functions (a) Schaffer (b) MMF1. (**b**) The Pareto front resulting from the MOMSA algorithm in multi-objective benchmark functions (a) Schaffer (b) MMF1. (**c**). The Pareto front resulting from the MOMGA algorithm in multi-objective benchmark functions (a) Schaffer (b) MMF1.

Regarding the metric of spacing (S), the MOAHA algorithm had a better distribution of non-dominant solutions (criterion S) in the studied benchmark functions. These results are more visible in Fig. 15.

Figure 15A–C show the Pareto front resulting from applying the MOAHA, MOMSA, and MOMGA algorithms in the Schaffer and MMF1 standard multi-objective benchmark functions.

Figure 15 displays a graphic comparison between the precise and calculated Pareto fronts using the MOAHA, MOMSA and MOMGA multi-objective algorithms to solve the studied multi-objective problems. The ideal Pareto front, optimal distribution, and acceptable distribution for non-dominated solutions were all successfully covered by the MOAHA multi-objective algorithm (Fig. 15). Based on Fig. 15 and Table 3, the performance of all the utilized algorithms was satisfactory. All three algorithms yielded suitable Pareto fronts in terms of coverage and diversity.

### A multi-objective simulation–optimization model of the Karun Basin reservoir system

The recently developed MOAHA, MOMSA, and MOMGA algorithms were used to solve the multi-objective simulation–optimization model of the Karun basin reservoir system for a long-term period of 15 years (2000–2015). Table 4 presents the values of multi-objective algorithms setting parameters for Karun multi-objective multi-reservoir system operation. This table provides the best values of multi-objective algorithms' parameters based on the sensitivity analysis. Furthermore, the population size, the number of iterations, and the Pareto front archive size were considered the same for a fair comparison.

**Table 4:** Values of multi-objective algorithms parameters for Karun multi-objective multi-reservoir system.

MOAHA	Iterations	Number of variables	Pareto front archive size	Size of population	–
	100	1800	500	500	–
MOMSA	Iterations	Number of variables	Pareto front archive size	Number of search agents	Number of pathfinders
	100	1800	500	500	100
MOMGA	Iterations	Number of variables	Pareto front archive size	Number of adaptive grid	–
	100	1800	500	500	–

Three objective functions were established for this model: minimizing the overall shortage in the downstream demands supply, reducing the overall discrepancy between reservoir volume and the volume required for flood control, and maximizing the total output power of hydropower plants (minimizing the total difference between the production power and the installed capacity of the power plant). These objective functions usually have an inverse relationship with each other; therefore, to achieve one of the objectives, one must inevitably stray from the other objectives. This problem can be resolved to some degree by using multi-objective optimization techniques and identifying the mode that best suits all the objectives.

The top values of the MOAHA, MOMSA, and MOMGA algorithms for objective functions corresponding to the Pareto front are displayed in Table 5.

**Table 5:** The objective functions that correlate to the Pareto front as a result of the examined methods.

MOMGA			MOMSA			MOAHA			Pareto front No
OF3	OF2	OF1	OF3	OF2	OF1	OF3	OF2	OF1	
592.87	636.59	384.42	472.40	627.37	369.03	642.1488	610.8271	188.735	1
528.28	617.86	1679.71	475.86	640.38	380.20	638.372	601.9697	288.2918	2
529.50	612.56	2779.33	487.63	635.36	479.23	626.7036	594.0727	1587.117	3
558.13	629.92	782.01	549.97	677.15	286.81	633.8203	589.73	687.3864	4
568.25	560.65	1382.24	547.92	679.65	286.86	636.5542	602.8733	488.0196	5
592.81	637.03	384.42	570.08	683.44	387.64	627.5982	597.0686	1087.156	6
607.86	628.46	285.43	660.91	692.44	295.25	638.7393	592.8084	587.7673	7
657.70	631.88	189.03	662.65	693.12	294.55	1636.394	587.4367	187.9897	8
704.76	563.03	690.49	662.38	694.73	193.90	675.505	592.072	591.9026	9
711.96	579.10	491.22	626.89	699.56	291.83	679.7486	588.7152	491.4279	10
714.82	577.71	491.35	647.53	699.89	193.04	679.3162	590.755	591.8041	11
717.08	589.34	392.17	629.74	700.57	292.19	665.6961	545.3682	1690.363	12
721.65	573.52	491.90	651.35	704.97	193.52	674.0022	548.0283	1390.303	13
724.02	576.31	292.07	691.56	707.85	196.86	674.4901	546.1013	1290.303	14
726.94	579.96	192.46	716.41	712.00	198.21	692.9982	575.1266	792.4417	15
727.95	570.35	292.09	719.43	716.12	198.39	665.0339	599.5215	389.5457	16
727.96	569.15	292.79	727.70	717.10	198.92	1658.245	577.5739	488.3745	17
1662.25	613.60	189.06	663.69	693.71	295.19	1658.674	554.3481	689.0188	18
1700.97	592.48	191.22	475.12	604.93	1977.73	677.3143	577.2137	691.2642	19
1716.94	562.33	491.56	451.70	607.15	2478.54	664.6564	601.1212	389.5457	20
1478.61			1468.80			1441.71			Minimum total objectives
3921.39			3537.3			2902.04			Maximum total objectives
15,479.01			14,970.13			13,345.16			CPU run time (s)

The Pareto front’s finest optimal point was chosen to minimize the sum of objective functions and managed to simultaneously bring all three objectives to the global optimum (Table 5). The MOAHA algorithm with OF1 = 188.73, OF2 = 610.83, OF3 = 642.15, and total objectives of 1441.71 had a better performance than the other two algorithms in the complex and large-scale problem of multi-objective optimal operation of the Karun basin reservoir system. After the MOAHA algorithm, the MOMSA algorithm with OF1 = 369.03, OF2 = 627.37, OF3 = 472.4 and total objectives of 1468.8, and the MOMGA algorithm with OF1 = 189.03, OF2 = 613.88, OF3 = 657.7 and total objectives of 1478.61 had the best results, respectively. The CPU run time for MOAHA was 13345s, while the value for MOMSA and MOMGA was 14970s and 15479s, respectively. This indicates that the MOAHA was the fastest algorithm in running the code achieving impressive results in a shorter time.

**Figure 16:**
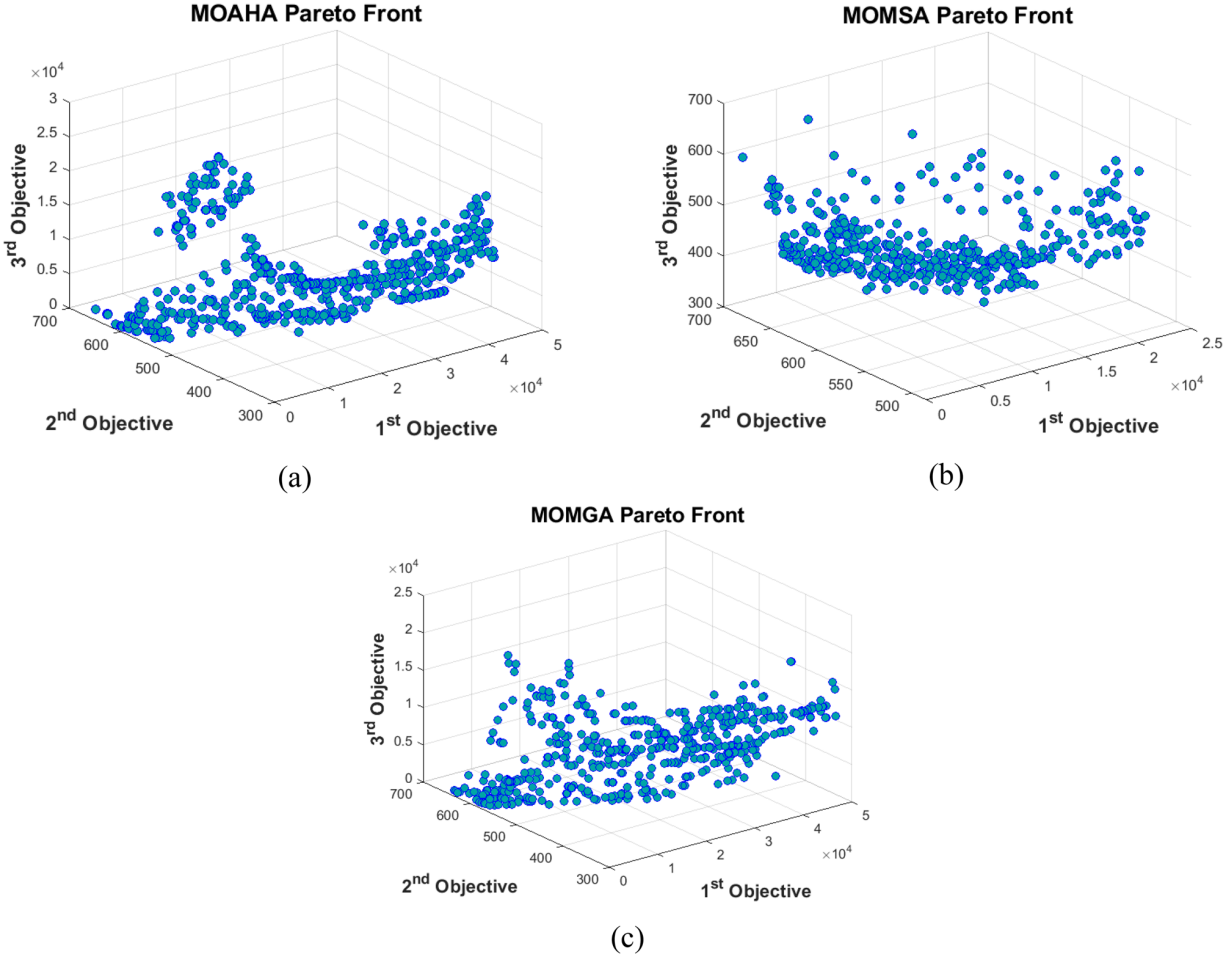
The Pareto front obtained from the implementation of different algorithms in the problem of multi-objective operation of the reservoir system of the Karun basin (**a**) MOAHA (**b**) MOMSA (**c**) MOMGA.

Figure 16 shows the Pareto front generated by the MOAHA, MOMSA, and MOMGA multi-objective algorithms in three dimensions from the values of the three objective functions (downstream demand–supply, hydropower energy production, and flood control).

Here, the closer the solution points are to the origin of the coordinates (the lower the Pareto front level), the more appropriate they are. Based on Fig. 16, the Pareto front for all three algorithms (MOAHA, MOMSA, and MOMGA) had an appropriate distribution of solutions around the Pareto front (had an outstanding quality in terms of coverage and diversity). As seen in the 3D views, the Pareto front obtained by the MOAHA algorithm has good diversity and coverage, which means all the non-dominated solutions were distributed uniformly along the Pareto front. This proves the high capability of this algorithm in simultaneously optimizing all three objective functions (OF1, OF2, OF3) of the Karun problem.

**Figure 17:**
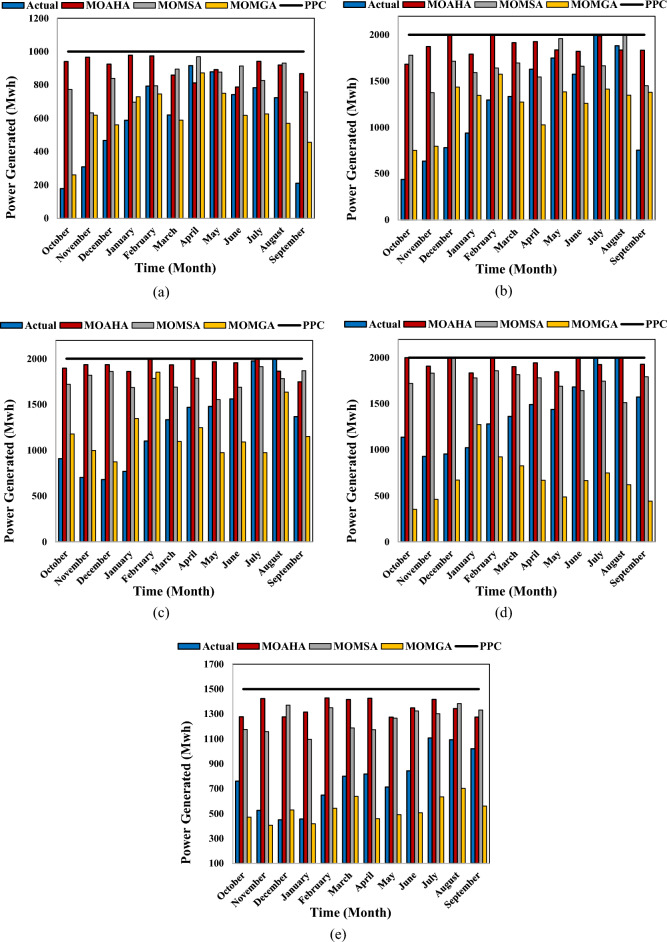
The energy produced in the studied reservoirs by different multi-objective algorithms in the long-term statistical period of 180 months (**a**) Karun 4 (**b**) Karun 3 (**c**) Karun 1 (**d**) Masjed-e-Soleyman (**e**) Gotvand Olia.

Figure 17 depicts the amounts of hydropower energy produced by the MOAHA, MOMSA, and MOMGA algorithms and actual exploitation conditions in the long-term statistical period of 180 months for Gotvand Olia, Masjed-e-Soleyman, Karun 1, Karun 3, and Karun 4 dams.

According to Fig. 17, the amount of energy produced by the MOAHA algorithm was higher than the other two algorithms, and the actual operating conditions were closer to the power plant's production capacity. The average energy produced by MOMSA, MOAHA, and MOMGA algorithms in the Karun basin reservoir system for 15 years was 17,166.47, 15,669.70, and 9026.31 GW per year, respectively, which is slightly better than the average actual exploitation in the 15 years (11,294.84 GW per year). The results indicate a 51.98% superiority of the MOAHA algorithm in hydropower energy production for the reservoir system of the Karun basin compared to the actual exploitation conditions.

The performance indicators of water systems are essential criteria in evaluating the accuracy of management methods of these systems. Table 6 presents the results of indicators used in multi-objective optimal exploitation management of the reservoir system of the Karun basin using the new MOAHA, MOMSA, and MOMGA algorithms and actual conditions exploitation.

**Table 6:** The results of performance indicators of water systems in multi-objective optimal exploitation management of the reservoir system of the Karun basin using the MOAHA, MOMSA and MOMGA algorithms.

Reservoir	Index	MOAHA	MOMSA	MOMGA	Actual
Karun4	Reliability	100	100	97.78	100
	Resilience	100	100	80	100
	Vulnerability	0	0	39.95	0
	Sustainability	100	100	77.74	100.00
Karun3	Reliability	100	100	98.33	99.41
	Resilience	100	100	100	100
	Vulnerability	0	0	26.56	58.62
	Sustainability	100	100	89.72	74.37
Karun1	Reliability	100	100	97.22	100
	Resilience	100	100	100	100
	Vulnerability	0.051	0.041	14.06	0.054
	Sustainability	99.98	99.99	94.19	99.98
Masjed-e-Soleyman	Reliability	100	100	98.89	100
	Resilience	100	100	100	100
	Vulnerability	0	0	30.39	0
	Sustainability	100	100	88.30	100.00
Gotvand Oliya	Reliability	58.89	72.78	21.11	51.04
	Resilience	52.65	73.56	22.38	25.86
	Vulnerability	72.89	92.89	69.43	93.92
	Sustainability	43.80	33.64	24.35	20.02
Average	Reliability	91.778	94.556	82.666	90.09
	Resilience	90.53	94.712	80.476	85.172
	Vulnerability	14.5882	18.5862	36.078	30.5188
	Sustainability	88.76	86.73	74.86	78.87

According to Table 6, the MOAHA algorithm ranked first among the applied algorithms with an average sustainability index of 88.76%. The average values of reliability, resiliency, and vulnerability for this algorithm were 91.78 (Rel), 90.53 (Res), and 14.59 (Vul), respectively. After that, the MOMSA algorithm (Rel = 94.56, Res = 94.71, Vul = 18.59, and Sus = 86.73%), followed by the actual exploitation (Rel = 90.09, Res = 85.17, Vul = 30.52, and Sus = 78.87%) and the MOMGA algorithm (Rel = 82.67, Res = 80.48, and Vul = 36.08, and Sus = 74.86%), ranked two to four, respectively. The new MOAHA algorithm has a significant advantage in managing the multi-objective exploitation of the Karun basin reservoir system.

Here, the results of this study are compared with those of similar studies on the optimal operation of reservoirs. Based on Table 7, two criteria (the increase in the total power generation by the utilized model and the sustainability index of the studied water resource system optimized by the models) were employed to compare the performance of the utilized models. Regarding the increase in the total power generation, the MOAHA and MOMSA models (developed in the present study) increased the total power generation by up to 50.78% and 37.37% compared to the actual exploitation conditions, respectively. Ahmadianfar et al.^31^ developed an SATLDE algorithm that could raise the total power generation by up to 23.70%. The performance of their algorithm was comparable with the performance of the MOMSA and MOMGA algorithms of the present study, but its performance is far from that of the MOAHA algorithm. Therefore, among the four algorithms applied for the optimal operation of reservoirs, the MOAHA was superior in terms of total power generation. Note that each algorithm was developed for a specific area, and this problem should be considered in the comparisons.

**Table 7:** Comparison of different algorithms in the optimal operation of reservoirs.

Criteria	References	Algorithm	Result (%)
Increasing the total power generation (%)	Ahmadianfar et al.^31^	SATLDE	23.70
	Current study	MOMSA	37.37
		MOAHA	50.78
Sustainability index (%)	Sharifi et al.^30^	NSGA-II	22.73
		SPEA-II	46.09
	Current study	MOMGA	74.86
		MOMSA	86.73
		MOAHA	88.76

In terms of the sustainability index, the best results were obtained by MOAHA (88.76%), MOMSA (86.73%), and MOMGA (74.86%) of the present study, and followed by the SPEA-II (46.09%) and NSGA-II (22.73%) developed by Sharifi et al.^55^.

## Conclusion

This study employed a multi-objective version of the MOAHA artificial hummingbird algorithm to solve complex and large-scale multi-objective problems. The results were compared with two novel multi-objective algorithms with proven results in numerous studies, i.e., MOMSA and MOMGA. Two standard multi-objective benchmark functions, Schaffer and MMF1, were used to check the efficiency of the novel multi-objective algorithms. The performance of the new multi-objective algorithms was also evaluated using four performance assessment criteria (GD, S, Δ, and MS). The multi-reservoir-multi-objective optimal operation problem for the Karun basin reservoir system was coded and modeled in MATLAB to minimize the total shortfall in supplying downstream needs, the total difference in reservoir volume from the required volume for flood control, and the total difference in production power from the installation capacity of the power plants. The uncertainties (or potential limitations) regarding the utilized data and the modeling procedure can be taken into consideration in future studies. Reservoir performance indices, including reliability, vulnerability, reversibility, and stability indices, were utilized to evaluate exploitation policies from different algorithms. The results of applying the investigated multi-objective algorithms in solving the standard Schaffer and MMF1 multi-objective problems showed that all three multi-objective algorithms successfully covered the optimal Pareto front and the optimal distribution for non-dominant solutions. Meanwhile, the MOAHA multi-objective algorithm had the best performance. The results of the effectiveness of these algorithms in solving the complex and large-scale problem of multi-reservoir-multi-objective optimal exploitation of Karun basin reservoir system demonstrated the superiority of the MOAHA algorithm with an increase in the problem's size and complexity (Karun 4, Karun 3, Karun 1, Masjid Soleyman, and Gotvand Olia dams) in a long-term period of 180 months with 1800 decision variables, indicating the acceptable results of this algorithm. The MOAHA algorithm performed well with OF1 = 188.73, OF2 = 610.83, OF3 = 642.15, and a minimum of 1441.71 objectives. Considering the importance of hydropower energy production in hydropower systems, maximizing hydropower energy production, which was one of the key objectives in the Karun basin simulation optimization model, was analyzed using the algorithms. The findings demonstrated that the MOAHA algorithm was competent as it produced 17,166.47 GW of energy compared to the MOMSA, MOMGA, and actual exploitation conditions (which produced 15,669.70, 9026.31, and 11,294.84 GW of energy annually, respectively) for the Karun basin reservoir system over the 180 months (from September 2000 to August 2015). The CPU run time of the algorithms, which is an important criterion, was also studied. The CPU run time for the MOAHA was 13345s, while the MOMSA and MOMGA values were 14970s and 15479s, respectively. This indicates that the MOAHA was the fastest algorithm in running the code and achieved impressive results in a shorter time. Finally, the performance indicators of water systems, which are essential criteria in evaluating the accuracy of management methods of these systems, showed that the average sustainability index obtained from MOAHA, MOMSA, and MOMGA in the Karun basin reservoir system was 88.76, 86.73, and 74.86, respectively; these values suggest the acceptable performance of the MOAHA algorithm in managing the multi-objective exploitation of the Karun basin reservoir system. Given the new MOAHA algorithm's strong performance and capability as a contemporary multi-objective optimization method in various optimization problems, researchers are advised to use the mentioned algorithm for problems related to hydrology and water resources, particularly multi-reservoir multi-purpose systems.

## Data Availability

The datasets used and/or analysed during the current study available from the corresponding author on reasonable request.
